# Protein evolution analysis of *S*-hydroxynitrile lyase by complete sequence design utilizing the INTMSAlign software

**DOI:** 10.1038/srep08193

**Published:** 2015-02-03

**Authors:** Shogo Nakano, Yasuhisa Asano

**Affiliations:** 1 Biotechnology Research Center and Department of Biotechnology, Toyama Prefectural University, 5180 Kurokawa, Imizu, Toyama 939-0398, Japan; 2 Asano Active Enzyme Molecule Project, ERATO, JST, 5180 Kurokawa, Imizu, Toyama 939-0398, Japan

## Abstract

Development of software and methods for design of complete sequences of functional proteins could contribute to studies of protein engineering and protein evolution. To this end, we developed the INTMSAlign software, and used it to design functional proteins and evaluate their usefulness. The software could assign both consensus and correlation residues of target proteins. We generated three protein sequences with *S*-selective hydroxynitrile lyase (*S*-HNL) activity, which we call designed *S*-HNLs; these proteins folded as efficiently as the native *S*-HNL. Sequence and biochemical analysis of the designed *S*-HNLs suggested that accumulation of neutral mutations occurs during the process of *S*-HNLs evolution from a low-activity form to a high-activity (native) form. Taken together, our results demonstrate that our software and the associated methods could be applied not only to design of complete sequences, but also to predictions of protein evolution, especially within families such as esterases and *S*-HNLs.

Development of enzyme design methods could contribute to improvement and alteration of various aspects of enzyme function, such as stability and reactivity. To date, several methods for enzyme design have been reported, including ancestral sequence design^1^ and structure-based design^2^. These methods can be categorized based on their underlying approaches. In this study, we classify methods for enzyme design either as “bottom-up” or “top-down” approaches, as described by Blaber *et al*^3^. In the bottom-up approach, the desired activity is determined first, and then amino-acid substitution is repeatedly performed to obtain the desired activity^3^. This approach has been used to design nitric oxide reductase, designed by mutating myoglobin^4^,^5^, as well as Kemp eliminase^2^,^6^. In the top-down approach, on the other hand, the sequences of functional proteins are designed first, and then their functionally important residues are predicted from biochemical analysis^3^. Within the top-down approach, consensus design is a representative method for creating complete sequences of functional proteins^3^. One advantage of this method is that both protein engineering and consideration of protein evolution can be performed at once^3^,^7^. In this study, we developed software and methods for consensus design of complete sequence.

To perform consensus design, one must first assign the consensus residues, i.e., the residues that appear most commonly at each position in the aligned sequences of proteins in the family of interest^8^,^9^,^10^. Here, the sequences used to assign the consensus residues are called the “library”. The libraries used to date contain dozens to thousands of sequences. After these assignments are completed, primary sequence of full-consensus proteins can be designed by selecting fully consensus residues. Some of the resultant full-consensus proteins have extraordinary properties relative to the native proteins, e.g., high thermal stability^11^ or refoldability from thermal denaturation^12^. Therefore, creation and biochemical analysis of full-consensus proteins may reveal the source of a protein's extraordinary properties. These features are useful in both protein engineering and prediction of protein evolution.

Although designing full-consensus proteins has certain advantages, creation of these proteins requires overcoming several challenges. First, the library should be prepared by selecting large numbers of sequences that are not phylogenetically biased, because biased libraries often generate proteins with low activities relative to the native proteins^9^,^12^. Curation, which is a procedure for removing unnecessary sequences from the library, is sometimes required in order to prepare non-biased sequences^12^. Second, the correlation residues, which often form unique interactions within each family, must be assigned from the library^13^. The frequencies (i.e., the rates of appearance) of correlation residues are perturbed correlatively when multiple sequence alignment (MSA) is performed by changing the combination of sequences^14^,^15^. As with consensus residues, assignment of correlation residues is important for the design of functional proteins^14^,^16^. However, this assignment is difficult, because correlation residues are often less conserved and cannot be revealed by conventional MSA methods.

As mentioned above, assignment of consensus and correlation residues is necessary for design of functional proteins. To date, however, few algorithms capable of finding these residues have been reported. To address this need, we developed the MSA software INTMSAlign. We then investigated whether the program could assign these residues in sequences of the α/β hydrolase fold superfamily, which includes esterases and *S-*selective hydroxynitrile lyases (*S-*HNLs). *S*-HNLs catalyze the degradation/synthesis of cyanohydrins, including (*S*)-mandelonitrile, to/from aldehyde and cyanide ion^17^. *S-*HNL is suitable as a target protein for validation of INTMSAlign for several reasons: i) *S*-HNL sequences could not be designed without curation, because the library contained more sequences of esterases than *S-*HNLs. ii) Many structural and biochemical analyses of *S-*HNLs have been performed previously^18^,^19^,^20^,^21^,^22^. Therefore, the function of all residues could be predicted accurately, in order to determine whether INTMSAlign assigned them correctly. Moreover, *S*-HNL is also an industrially useful protein^23^,^24^, providing another justification for selecting it as the target protein. After completing the validation, we prepared three complete sequences of designed proteins, HNL85, HNL54 and HNL30, using an INTMSAlign-based method called hybrid full-consensus sequence design (HyFSD). The designed proteins were intended to have *S*-HNL activity; therefore, we refer to them as designed *S*-HNLs. We analyzed the biochemical functions of the designed *S*-HNLs by measurement of CD spectra and enzyme kinetics. Our results show that INTMSAlign could assign both consensus and correlation residues, and that this software could be applied to the design of functional proteins. Finally, we will consider the protein evolution of the *S*-HNLs based on experimental data obtained from our designed *S-*HNLs and previous researches performed by other groups^25^,^26^.

## Results

### The INTMSAlign algorithm: Assignment of consensus residues

**Figure 1:**
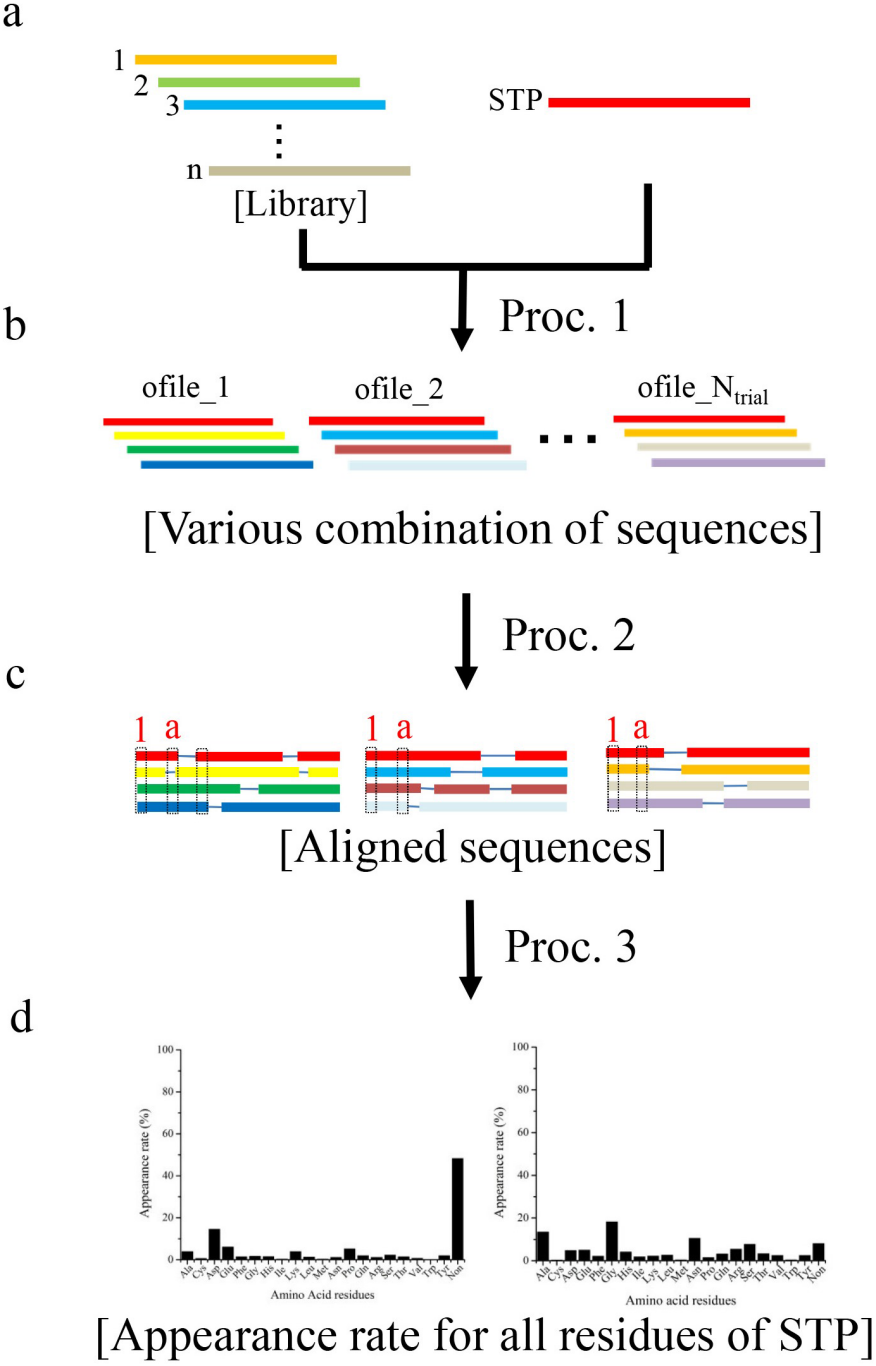
Schematic model of the INTMSAlign algorithm. Sequence of target protein (STP) and library, which contains hundreds to thousands of sequences from the STP family, must be prepared before the INTMSAlign algorithm is used (a). After the preparation of the library, *N*_trial_ ofiles are created by selecting one STP (red) and *N*_pick_ sequences from the library (proc. 1). Here, the STP is contained in the ofile (b). Next, multiple sequence alignment is applied to all of the ofiles using ClustalW (proc. 2), and *N*_trial_ of the aligned sequences are prepared (c). Finally, for *N*_trial_ aligned sequences, the number of all 20 amino-acid residues and gaps is summed up (dotted square) for every STP residue (red color letter in c). Finally, the frequencies are calculated (d).

A schematic view of the INTMSAlign algorithm is shown in Figure 1, and a more detailed description of the algorithm is provided in the supporting information (Supplementary Figures 1 and 2). INTMSAlign requires two files to run: the sequence of the target protein (STP) and a library consisting of sequences of proteins in the same family. The STP contains only one sequence, whereas the library contains a total of “n” sequences; there is no limit to the number “n” (Fig. 1). INTMSAlign can assign consensus residues for the STP via following three procedures. 1). A total of *N*_trial_ ofiles are created. The ofile is the input file for INTMSAlign; each file contains one STP and *N*_pick_ sequences selected randomly from the library (Fig. 1, proc 1). 2), MSA is applied to all ofiles using CLUSTALW (Fig. 1, proc 2). 3), Frequencies of amino-acid residues in the library are calculated for each position in the STP (Fig. 1, proc 3). The STP provides the basis for calculating the frequencies; therefore, the STP is always contained in each file (Fig. 1b). Utilizing INTMSAlign, consensus residues can theoretically be assigned for an unlimited number of sequences contained in the library at once; this is the biggest advantage of INTMSAlign relative to other MSA programs, which limit the available number of sequences^27^. The sequences in the library share more than 20% sequence identity with the STP, and INTMSAlign utilizes CLUSTALW to align the sequences. To assign consensus residues correctly, *N*_trial_ must grow as the number of sequences in the library increases.

In this study, the sequence of *S*-HNL from *Manihot esculenta* (*Me*HNL) was used as the STP, and the consensus residues were extracted from the library. *Me*HNL is a native *S*-HNL, and many functional analyses of this protein have been performed previously. *S*-HNL, which belongs to the α/β hydrolase fold superfamily, catalyzes addition and elimination of a nitrile group from its substrates^28^,^29^. The superfamily includes esterases, which catalyze hydrolysis of various esters^30^. The sequence identity between *Me*HNL and the esterases is relatively high: for example, the identity between *Me*HNL and salicylic acid binding protein 2 (SABP2) esterase from tobacco^30^, is 40.3% (Table 1).

The runtime parameters of INTMSAlign are shown in Supplementary Table 1; there are a total 823 sequences in the library, which were obtained by submitting sequences of *Me*HNL to the Blastp web server (Supplementary Table 1). Because the number of proteins in the family will increase in the future, the appearance rate shown in this study should change as the number of sequences increases. The number of CLUSTALW processes (*N_trial_*) was 1000, and the number of sequences extracted from the library (*N_pick_*) was 8 (Supplementary Table 1). From this analysis, it was clear that the program could classify enzymatically important residues of *Me*HNL (Ser80 and His236) as consensus residues (Supplementary Fig. 3a, b).

**Figure 2:**
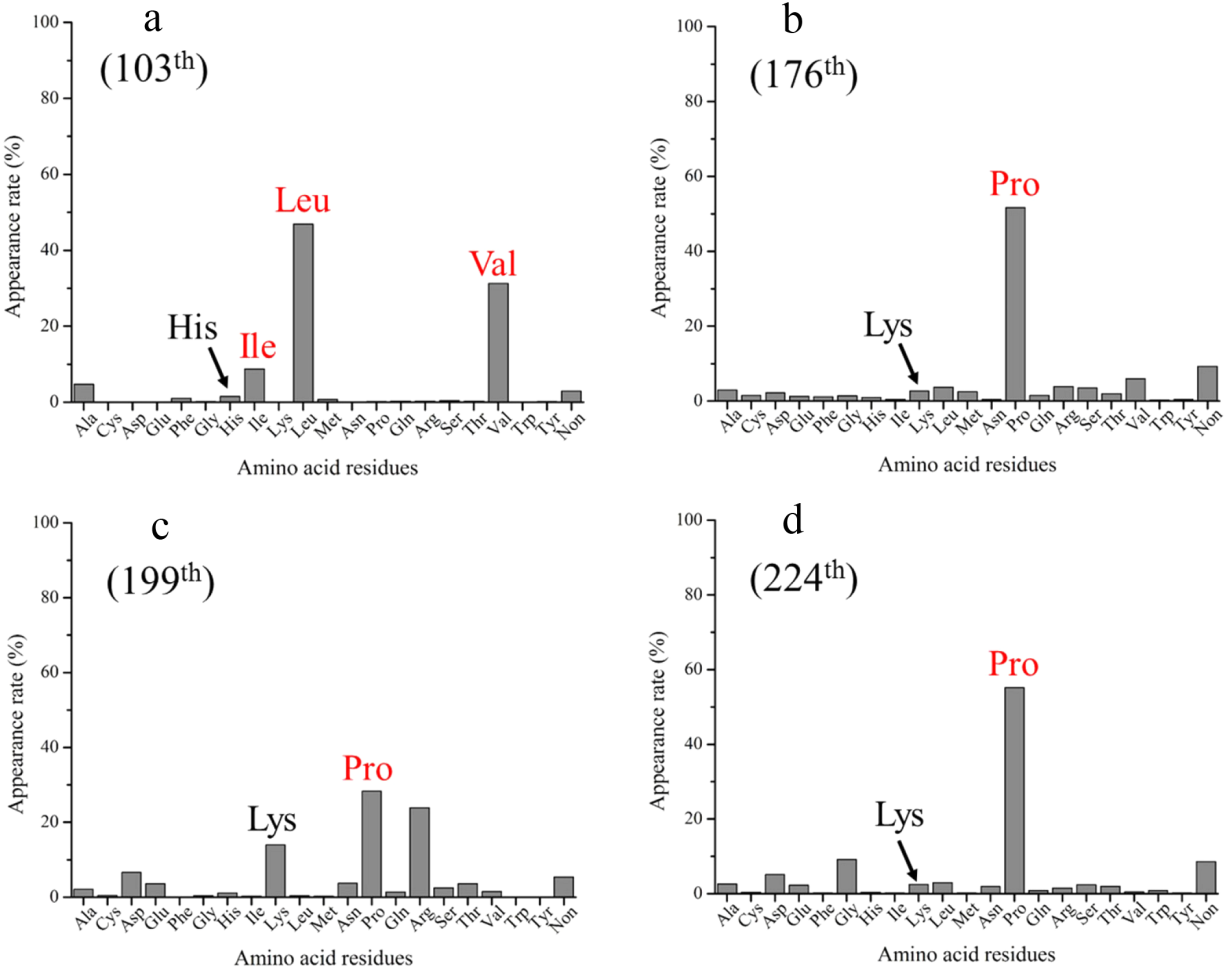
The frequencies of His103 (a), Lys176 (b), Lys199 (c) and Lys224 (d) of *Me*HNL. Four residues [103, 176, 199, and 224, ref. 20] that improved the solubility of *Me*HNL in the *E. coli* expression system are colored in red.

At this point, an important question arises: what is the advantage of assigning consensus residues when we perform protein engineering? To illustrate the advantage of such assignments, we investigated the consensus residues at positions 103, 176, 199, and 224 of *Me*HNL. In a previous study, Asano *et al.* showed that mutation of these residues could improve the solubility of *Me*HNL in an *E. coli* expression system^20^. The frequencies of these residues are shown in Fig. 2; the consensus residues at positions 103, 176, 199, and 224 are Leu, Pro, Pro, and Pro, respectively. Surprisingly, all of these consensus residues were in accordance with residues in which mutations can improve the solubility of *Me*HNL^20^. These results imply that proteins that are insoluble in an *E. coli* expression system can be solubilized by mutating certain residues to the consensus residues.

Collectively, these results confirm that INTMSAlign can assign consensus residues.

### The “residue fixation” algorithm implemented in INTMSAlign: Assignment of correlation residues

Sequences belonging to families other than that of the STP were frequently included in INTMSAlign libraries, and the existence of such sequences made it difficult to assign consensus residues from a single family. Curation of the library is necessary in order to resolve this difficulty; therefore, a function called “residue fixation” was implemented to INTMSAlign (Supplementary Fig. 4). An overview of this function is as follows: initially, users define a “marker residue” to perform curation of the library. Residue numbers and kinds of amino-acid residues are utilized in residue fixation; in Supplementary Fig. 4, these are defined as “2:T”. After the definition is complete, INTMSAlign selects sequences in the library that have the same residue at position as that of marker residue, and then calculates the appearance rate (Supplementary Fig. 4). In case of “2:T”, INTMSAlign selects sequences from the library with Thr at the second position and calculates the frequency (i.e., the appearance rate), shown in red in Supplementary Fig. 4. Detailed functional explanation is provided in the supporting information.

We will now describe an example application of the residue fixation function. In the case of *Me*HNL, enzyme sequences from at least two families were present in the library: esterases and *S-*HNLs. In this case, the library contains more esterase sequences than *S*-HNL sequences, i.e., the library is phylogenically biased toward esterases. Thus, curation of the library is required to assign consensus residues specific to the *S-*HNL family. A previous study chose position 237 as the marker residue; this amino acid is Met in esterases and Lys in *S-*HNLs^26^. By utilizing the marker residue, two result files could be prepared: one was obtained by utilizing only sequences of esterases (condition of residue fixation, 237:M) in the library, and the other was obtained by utilizing only sequences of *S*-HNLs (condition of residue fixation, 237:K). From comparison of both files, we detected five residues that are individually conserved in esterases and *S-*HNLs (Supplementary Fig. 5 and Supplementary Table 2). Three of the five identified residues form hydrogen-bond interactions with each other in *Me*HNL (Supplementary Figure 6a). A quantum-mechanical calculation has revealed that this interaction is important for catalysis by *S*-HNLs^31^. According to a previous definition^16^, these residues could be regarded as correlation residues.

Based on these results, we confirmed that correlation residues can be extracted by applying the residue fixation method with a defined marker residue. This finding also indicated that curation of the library can be performed by utilizing marker residues. This curation method would be effective for handling libraries containing large numbers of sequences. By contrast, when using normal MSA methods alone, it is hard to separate very large number of sequences into different groups after completion of the MSA.

### Hybrid full-consensus sequence design of complete sequences of designed *S-*HNLs

**Figure 3:**
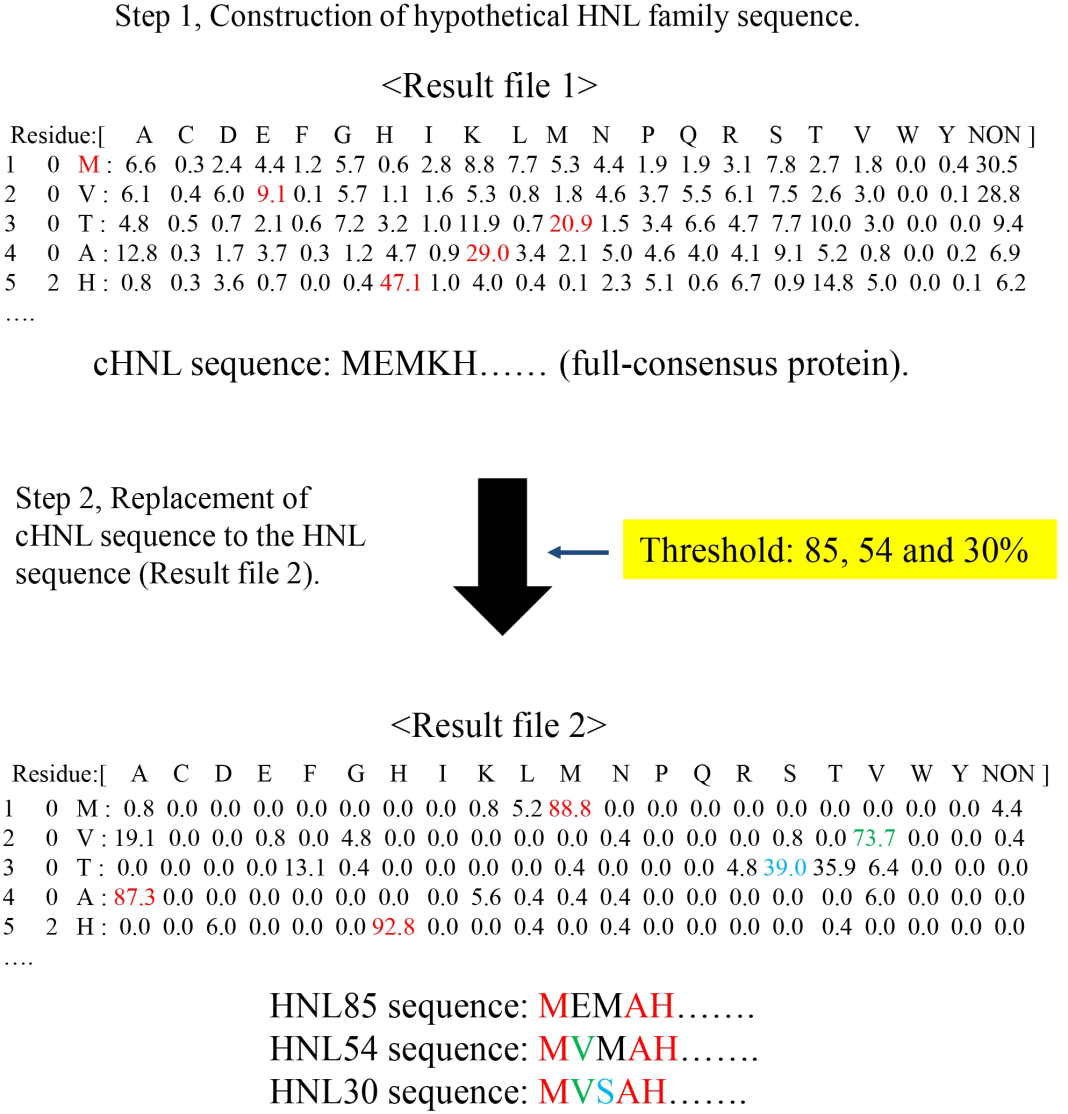
Hybrid full-consensus sequence design (HyFSD) method for generating designed *S*-HNLs. Before designing the sequence, two output files were prepared by INTMSAlign: “Result file 1” and “Result file 2”, prepared without and with residue fixation, respectively. In the case of the *S-*HNLs, the residue fixation condition was set to 237:K (i.e., only sequences in the library for which the 237^th^ residue was Lys were utilized to calculate the frequencies). The resultant hybrid full-consensus sequences were named based on their difference from the threshold value; HNL85, HNL54, and HNL30.

We designed complete sequences of designed *S*-HNLs using a technique based on INTMSAlign: hybrid full-consensus sequence design (HyFSD method, Fig. 3). Two result files, Result file 1 and Result file 2, were required to create sequences using the HyFSD method (Fig. 3). After preparation of the result files, the new sequences were created via the following steps: i) The sequence of the full-consensus protein (cHNL in Fig. 3) was generated by selecting consensus residues from Result file 1 (shown as red color, Fig. 3), ii) Next, residues in the cHNL sequence was replaced with the corresponding consensus residues of Result file 2, which was prepared by applying residue fixation (237:K). Here, Result file 2 represents the consensus residues specific to *S*-HNLs (Fig. 3). The replacement was performed when the frequency of the consensus residues was higher than a user-defined threshold value (yellow filled square in Fig. 3).

In this study, we created complete sequences of three proteins using the HyFSD method: HNL85, HNL54 and HNL30, which we call designed *S*-HNLs. Initially, the number in the sequence name represented the threshold value (Fig. 3). A total of 100 (HNL85), 158 (HNL54), and 258 (HNL30) sequences of cHNL are substituted for the consensus sequence in Result file 2 (Fig. 3): HNL30 consists entirely of the *S*-HNL consensus sequence. The sequences of these designed *S-*HNLs should be similar to that of *Me*HNL (Table 1); the sequences of HNL30 and HNL85 are the most similar to *Me*HNL and SABP2, respectively (Table 1). Blastp search revealed no natural proteins identical to the designed *S*-HNLs.

A phylogenetic tree was built utilizing 11 sequences of the α/β hydrolase fold family and our designed *S*-HNLs (Supplementary Fig. 8A and B). As judged from bootstrap analysis, the tree could be divided into following three clades: esterase, unknown, and *S*-HNL family (Supplementary Fig. 8A and B). HNL30 and HNL54 belong to the *S*-HNL family clade, and HNL54 is close to an ancestor of the family (Supplementary Fig. 8A and B).

### Analysis of protein expression, folding, and thermal stability of the designed *S-*HNLs

Three of designed *S-*HNLs (HNL85, HNL54 and HNL30) could be expressed in *E. coli*. HNL85 and HNL54 were expressed in the soluble fraction, and more than 5 mg of proteins were obtained from 1 L of culture. On the other hand, the solubility of HNL30 was lower than that of the other two designed *S-*HNLs: less than 0.5 mg of the protein could be obtained from 1 L of culture.

**Figure 4:**
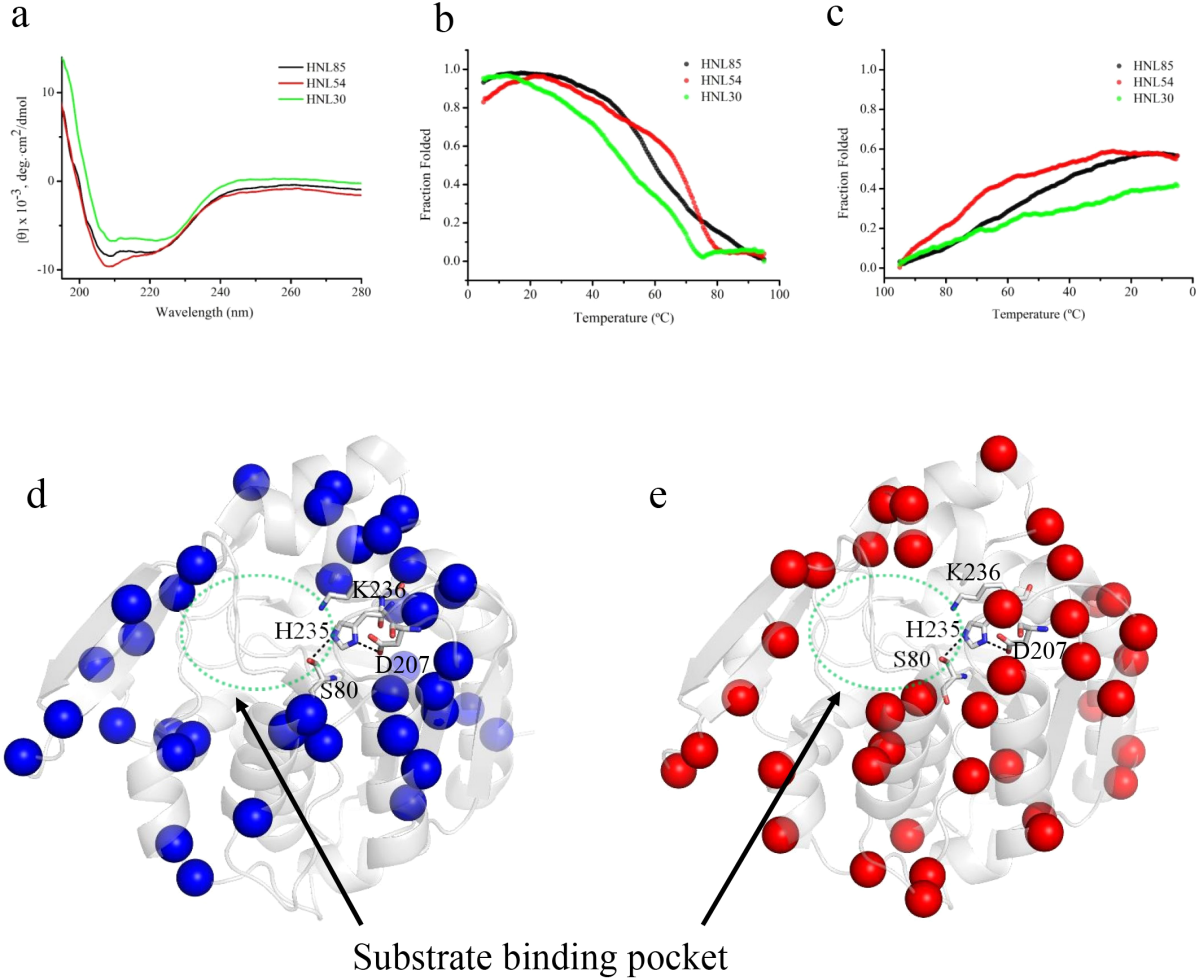
(a) CD wavelength spectra of three designed *S*-HNLs. Spectra of HNL85, HNL54, and HNL30 are colored in black, red and blue line, respectively. (b) Thermal denaturation and (c) renaturation of three designed *S*-HNLs. The color of the lines is the same as those of Figure 4a. (d–e) Representation of mutation sites on the *Me*HNL structure (PDB ID: 1EB9). Based on the result of the MSA (Supplementary Fig. 7), Cα atoms of sites mutated HNL85 and HNL54 (blue spheres, 4d) or between HNL54 and HNL30 (red spheres, 4e) were selected and shown as spheres in the *Me*HNL structure.

CD spectra of the three designed *S-*HNLs were measured to confirm whether the *S*-HNLs could fold correctly (Fig. 4a), as even very soluble proteins are sometimes unfolded^32^. The spectra indicated that the designed *S-*HNLs, like native *S-*HNLs, have an α/β hydrolase fold. In fact, the spectral features of the designed *S*-HNLs (Fig. 4a) correspond to the CD spectrum of *Me*HNL, a native *S-*HNL^20^.

To assess the thermostability of the designed *S-*HNLs, we measured thermal denaturation by monitoring the spectral change at 222 nm (Fig. 4b). Among the designed *S*-HNLs, HNL54 had the highest thermal stability, and HNL30 had the lowest; the midpoints of the cooperative transition temperatures (*T*_m_) of HNL85, HNL54, and HNL30 were 60, 67.5, and 50.5°C, respectively. Thermal renaturation also occurred in the designed *S*-HNLs; about 60% (HNL85 and HNL54) and 40% (HNL30) of the protein were refolded by lowering temperature (Fig. 4c).

### Enzyme kinetic analysis of the designed *S-*HNLs

We then analyzed the enzyme kinetic parameters of the three designed *S*-HNLs, using *rac*-Man as a substrate (Table 2). The catalytic efficiency of the designed *S*-HNLs increased along with their sequence identity to *Me*HNL. The parameters of HNL85 could not be determined because of its low activity. The catalytic efficiencies (*k*_cat_/*K*_m_) of HNL54 and HNL30 were, respectively, about 0.01- and 0.5-fold of that of *Me*HNL (Table 2).

Next, we estimated the enantioselectivity of the designed *S*-HNLs by utilizing (*R*)- and (*S*)-Man as substrates (Table 3). The designed *S*-HNLs exhibited *S*-selectivity, and the selectivity of HNL30 was higher than that of HNL54 and HNL85. Although the parameters of HNL85 could not be determined because of its low activity (Table 3), analysis of the synthetic reaction revealed that HNL85 exhibited *S*-selectivity: the peak area of (*S*)-Man grew as a function of the amount of enzyme (Supplementary Fig. 9a). HNL54 had weak *S*-selectivity: the *E*-value was 4.9 (Table 3), and it could synthesize both (*R*)- and (*S*)-Man (Supplementary Fig. 9b). Among the three designed *S*-HNLs, HNL54 had the highest catalytic efficiency toward (*R*)-Man (Table 3). The catalytic efficiency of HNL30 toward (*S*)-Man was more than 10-fold higher than that of HNL54 (Table 3), whereas the efficiency of HNL30 toward (*R*)-Man was about 0.5-fold of that of HNL54 (Table 3). Ultimately, this higher catalytic efficiency resulted in an increase of HNL30's *E*-value for (*S*)-Man (> 100, Table 3). The high *S*-selectivity of HNL30 was also confirmed in the synthetic reaction (Supplementary Fig. 9c).

Taken together, the level of *S*-HNL activity could be ranked in the following order: HNL85 < HNL54 < HNL30. Despite the presence of mutations in active-site residues specific to *S*-HNL, the *S*-HNL activity of HNL85 was too weak to determine the kinetic parameters (Table 2 and 3). In addition, the esterase activity of HNL85 could not be confirmed. These findings implied that mutation of highly correlated residues (Supplementary Table 2) could switch the protein's function between esterase and *S*-HNL. However, accumulation of mutations is still necessary in order to improve *S*-HNL activity. Consistent with this, Padhi *et al.* also suggested that additional mutations were necessary to improve *S*-HNL activity of SABP2 (G12T/M239K)^26^.

### Location of mutation site among the designed *S-*HNLs

Enzyme kinetics of the designed *S*-HNLs revealed that their catalytic efficiencies (*k*_cat_/*K*_m_) toward *rac*- and (*S*)-Man increased in the following order: HNL85, HNL54, and HNL30 (Table 2 and 3). Identification of the sites of mutations relative to the *S*-HNL structure would contribute to determining the factors that could improve the enzyme efficiency in the three designed *S*-HNLs. Therefore, we selected residues that were mutated between HNL85 and HNL54 (Fig. 4d, blue sphere) and between HNL54 and HNL30 (Fig. 4e, red sphere). These residues are shown on the structure of the native *S*-HNL *Me*HNL (PDB ID: 1EB9).

The comparative analysis of HNL85 and HNL54 indicated that the mutation sites were mainly located on the protein surface: of a total of 39 mutation sites, only one residue (106) was located within 10 Å of the Oγ atom of Ser80, which is a catalytic residue of *S*-HNL^22^. The average distance among the Oγ(Ser80) and Cα atoms of the mutation sites was high: 16.6 ± 5.0 Å. The comparison between HNL54 and HNL30 (Fig. 4e) yielded similar results: of a total 39 mutated residues, two (81 and 105) was located within 10 Å of the Oγ(Ser80) atom, and the average distance was also high: 17.1 ± 5.6 Å. In both cases, about 25% of the mutation sites are located at the substrate entrance region of *S*-HNLs (residues 115–147 and 178–186). Because flexibility and conformational change in this region are important for the substrate specificity of *S*-HNLs^21^, we predict that mutation at the region among the designed *S*-HNLs may affect their enzyme kinetics (Table 2 and 3). The crystal structures of the designed *S*-HNLs could help to predict differences in their kinetics; hence, structure determination of the designed *S*-HNLs is currently underway.

In other enzymes, such as simvastatin synthase^33^ and dihydrofolate reductase^34^, remote mutations often affect the dynamics of active-site residues and kinetics parameters. Likewise, in the designed *S*-HNLs, the remote locations of the mutation sites were confirmed. Therefore, localization and dynamic behavior during the reaction are the main factors that affect the enzyme kinetics of the designed *S*-HNLs.

## Discussion

**Figure 5:**
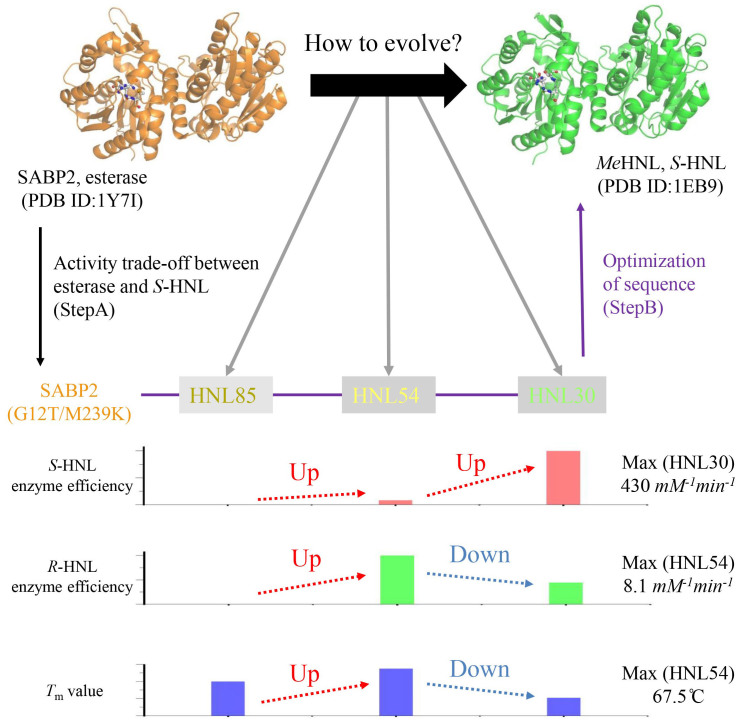
Schematic model of protein evolution for *S*-HNL. Based on previous studies and biochemical analysis of the designed *S*-HNLs, we hypothesized that esterase (SABP2) ultimately obtains native *S*-HNL (*Me*HNL) activity via Step A (black arrow) and Step B (purple arrow). The hypothesis is also supported by the high structural similarity between esterase and *S*-HNL: the rmsd value for Cα atoms between SABP2 (esterase) and *Me*HNL (*S*-HNL) was 0.751Å. Enzymatic efficiency of designed *S*-HNLs toward (*S*)-Man and (*R*)-Man was normalized, and is shown here as a red and blue bar graph. The *T*_m_ values (range: 40–70°C) of the designed *S*-HNLs are shown here by a blue bar graph.

In this study, we predicted protein evolution of *S*-HNLs from sequence and performed biochemical analysis of designed *S*-HNLs. These analyses demonstrate that the software we developed, INTMSAlign, can be utilized to predict evolution. The results of these predictions led us to postulate that the evolution of esterases ultimately resulted in native-like *S*-HNL activity. This hypothesis was based in part on the observation that both *S*-HNLs and esterases share high sequence and structural similarity with each other. Furthermore, in contrast to the *S*-HNLs, whose existence is only confirmed in certain cyanogenic plants, esterases with α/β hydrolase folds are present in a wide range of species^30^,^35^. Following this hypothesis and taking into account the phylogenetic tree (Supporting Fig. 8A and B), we speculate that the designed *S*-HNLs would evolve in the following order: HNL85, HNL54, HNL30. A schematic model of the predicted protein evolution of the *S*-HNLs is shown in Fig. 5.

It appears that an esterase can evolve into an *S*-HNL via the following steps: A) Trade-off between esterase activity and *S*-HNL activity (black arrow line in Fig. 5). B) Optimization of sequence to maximize *S*-HNL activity (purple arrow line in Fig. 5). Regarding step A, Padhi *et al.* proposed one mechanism by which an esterase could acquire *S*-HNL activity: after the esterase gene is duplicated, one of the copies can obtain *S*-HNL activity by mutating a few residues at the cost of its esterase activity^26^. Because their 11^th^ and 236^th^ residues were Thr and Lys, respectively, none of the designed *S*-HNLs were predicted to have esterase activity (asterisk, Supplementary Fig. 7); these residues would be catalytic in *S*-HNLs, but not in esterases. In fact, the trade-off from esterase to *S*-HNL activity occurred as a consequence of the G12T and M239K mutations in SABP2^26^ (asterisk, Supplementary Fig. 7). As expected, the esterase activity of HNL85 was too weak to determine the kinetic parameters. Although HNL85 had *S*-HNL activity, its activity and selectivity are much lower than those of native *S*-HNLs^26^. Therefore, step B must occur in order to confer native-like *S*-HNL activity on these proteins.

Step B can be predicted from the biochemical parameters of the designed *S*-HNLs (purple arrow line in Fig. 5). Initially, weak *S*-HNL activity is improved to moderate activity. During this process, activity toward (*R*)-Man would increase, as would the activity toward (*S*)-Man (Fig. 5). This process corresponds to protein evolution from HNL85 to HNL54. Subsequently, the activity toward (*S*)-Man is elevated. The second process corresponds to the evolution from HNL54 to HNL30. Here, a functional trade-off occurred during conversion from HNL54 to HNL30; *S*-HNL activity was improved by sacrificing enzymatic efficiency toward (*R*)-Man (Fig. 5). In addition, we also confirmed a trade-off in thermal stability: the *T*_m_ value of HNL54 was more than 15°C lower than that of HNL30 (Fig. 3).

During protein evolution from HNL85 to HNL30, mutations mainly occurred at positions remote from the active site, such as on the protein surface (Fig. 4d and 4E). This suggests that step B is achieved via accumulation of neutral mutations. Thus, relevant protein dynamics are required in order for *S*-HNLs to achieve high activity and selectivity. In general, enzymes attain new functions by mutating residues in the core regions, such as in step A, but the function is often quietly lower than that of native enzymes. In addition, the mutation often destabilizes the enzyme^36^. Therefore, accumulation of neutral mutations, which additively improve stability and dynamics, is necessary in order to accept the unfavorable mutation and thereby improve enzymatic function^37^,^38^. In the designed *S*-HNLs, HNL54 is the most stable, and is therefore most able to accommodate an unfavorable mutation in the core region.

As for the predicted model (Fig. 5), we recognize that this is one of several possibilities for protein evolution of *S*-HNL, as determined by the analysis of the designed *S*-HNLs. Alternatively, an ancestral esterase or *S*-HNL might exist, and it might attain esterase or *S*-HNL reactivity via various genetic processes, such as gene duplication. In addition, the predicted model (Fig. 5) was constructed by horizontal analysis, and the history of accumulation of mutations is not reflected to the model^39^,^40^. In the future, new hypotheses about evolution may be proposed by vertical analysis that accommodates the mutational history^39^, or by discovery and analysis of the ancestral *S*-HNL.

In the context of protein engineering, obtaining the hybrid proteins may contribute to improvements in the thermal stability of the target proteins. The thermal stability of the designed *S*-HNLs was high: the *T*_m_ of HNL54 is more than 15°C higher than that of HNL30. This suggested that some of the mutated residues in the designed *S*-HNLs improve the protein's thermal stability. Construction of chimeric enzymes of the designed and native *S*-HNL may be an effective means of creating *S*-HNLs with high thermal stability. Because these *S*-HNLs share high sequence identify each other (Table 1), we could obtain such chimeric *S*-HNLs using a directed evolution method such as DNA shuffling^41^.

In this study, we succeeded in extracting both consensus and correlation residues of *S*-HNLs using the INTMSAlign software. Furthermore, we were able to design complete sequences of designed *S*-HNLs using the HyFSD method based on INTMSAlign. The successful design of designed *S*-HNLs provides evidence that INTMSAlign could assign these residues accurately. Consistent with this, another group reported that foldable proteins can be designed when both consensus and correlation residues are correctly assigned^14^,^16^. The method we developed could design designed proteins that represent evolutionary intermediates between two families, a remarkable advantage relative to other design methods. Thus, protein evolution may be predicted through design and experimental analysis of the designed proteins, as shown in the case of the designed *S*-HNLs (Fig. 5). INTMSAlign only provides simple data, i.e., frequencies of amino-acid residues in the library. However, in several situations, these data may be sufficient for researchers who are planning to perform protein engineering: such individuals know more than anyone else about handling their target protein, and could therefore could obtain useful information from simple data, as in the case of the designed *S*-HNLs described in this study.

Although we have focused on describing the merits of INTMSAlign, we acknowledge that the software also has weaknesses. First, the output of the INTMSAlign is strongly dependent on the input data, especially the library: the method highlights the frequencies of residues belonging to the major group in the family, so residue fixation must be applied in order to obtain the frequencies from minor groups in the library. Changing the preparation method of the library is also an effective way to reduce bias: it is preferable to download the sequences from the PubMed web server directly by inputting keywords, such as enzyme numbers and protein names. Secondary, users have to manually find a “marker residue” by referring to various types of information, such as 3D structures and parameters of biochemical assays, prior to residue fixation. We are currently considering development of a new function that finds the marker residue automatically using only information contained in the library.

As illustrated by the design of designed *S*-HNLs, the INTMSAlign software and the derived HyFSD can be utilized for studies of protein engineering and protein evolution. Because these methods require only primary sequences, they could be applied not only to *S*-HNLs but also to various other proteins. For now, we are developing other applications of INTMSAlign to perform tasks such as screening of new enzymatic activities from primary sequence databases and identifying residues that could be point-mutated to improve protein function.

## Methods

### Overview of software, INTMSAlign

The INTegration of Multiple Sequence Alignment (INTMSAlign) software was developed to assign both consensus and correlation residues. INTMSAlign is derived from the well-known MSA program CLUSTALW^42^. The source code of INTMSAlign was written in the scripting language Python, and the iterative process of CLUSTALW was controlled by a shell script. The graphical user interface (GUI) of the INTMSAlign was written in wxPython. INTMSAlign is available from the corresponding author upon request via email. A web site for downloading the software is currently under construction.

### Cloning, expression, and purification of HNL85, HNL54, and HNL30

The *hnl85* gene encoding designed *S*-HNL (HNL85) was synthesized and ligated to plasmid pIDSMART by Integrated DNA Technologies (Coralville, IA, USA). The resultant plasmid, pIDSMART-hnl85, was digested with *Nde*I and *Xho*I and electrophoresed; the digested *hnl*85 gene was extracted from the agarose gel and ligated into the expression vector pET28b (Novagen, Madison, WI, USA). This plasmid was used to transform *E. coli* strain BL21(DE3). Transformants were cultivated for 4–5 hrs at 37°C in 1 L of LB medium supplemented with 30 μg/mL of kanamycin. To induce protein expression, 1.0 mM isopropyl-β-D-thio-galactopyranoside (IPTG) was added to the broth, and the culture was grown for an additional 24 hrs at 20°C.

After cultivation, cells were collected and suspended in buffer A (20 mM potassium phosphate [pH 7.0] and 100 mM NaCl) containing 10 mM imidazole, disrupted by sonication, and centrifuged. The supernatant was loaded onto a Ni-Sepharose column, and the column was washed with 50 ml of buffer A containing 70 mM imidazole. The sample was then eluted with buffer A containing 300 mM imidazole. The eluate was purified on a gel-filtration column (Superdex 75pg, GE Healthcare, Stockholm, Sweden) using buffer B (10 mM potassium phosphate [pH 7.0] containing 50 mM NaCl) as the loading buffer. All purification procedures were performed at 4°C. The purity of the sample was checked by SDS-PAGE. Protein concentration was determined by monitoring absorbance at 280 nm. An identical procedure was applied to the other designed *S-*HNLs (HNL54 and HNL30).

### HNL activity measurement

Initially, HNL activity was measured by monitoring benzaldehyde formed by degradation of mandelonitrile, e.g., racemic, (*R*)*-,* or (*S*)*-*mandelonitrile (*rac*-, [*R*]- and [*S*]-Man). Reaction mixture (100 mM citrate buffer [pH 5.5, 0.5–5 mM *rac*- or (*R*)- or (*S*)-Man) was incubated at 30°C for 30 min, and then transferred to a cuvette. After the enzyme solution was added to the cuvette, measurement of time-dependent absorbance change at 280 nm using a UV-2600 UV-Vis spectrometer (Shimadzu, Kyoto, Japan) was initiated immediately and proceeded for 1.5 min. The production rate of benzaldehyde (ε_280_ = 13800 M^−1^ cm^−1^) was calculated using the ORIGIN software (OriginLab, Northampton, MA, USA). All experiments were conducted in triplicate.

Second, we analyzed the cyanohydrins synthesis reaction. Activity was measured by monitoring synthesis of (*R*)- and (*S*)-Man. One hundred microliters of purified *S-*HNL was added to 900 μl of reaction mixture consisting of 300 mM citrate (pH 4.2), 100 mM KCN, and 50 mM benzaldehyde, and the mandelonitrile synthesis reaction was allowed to proceed at 30°C for 5 min. After the reaction was complete, 100 μl of the reaction mixture was removed and added to 900 μl of organic solvent (n-Hexane:2-Propanol = 85:15). Ten microliters of the solvent was then applied to a CHIRALCEL OJ-H column equipped on a the UFLC Prominence Liquid Chromatograph LC-20AD HPLC system (Shimadzu, Kyoto, Japan), and the UV-absorbance at 254 nm was monitored. The retention times for benzaldehyde and (*R*)- and (*S*)-Man were about 5.4, 11.5, and 14.4 min, respectively. The details of the experimental method were described previously^20^,^43^.

### Circular Dichroism spectroscopy

Circular dichroism (CD) spectra of the designed *S*-HNLs were measured utilizing a Jasco J-715 CD spectrometer. The buffer contained 10 mM potassium phosphate (pH 7.0) and 50 mM NaCl, and 0.06 mg/ml of purified designed *S-*HNLs was utilized in the measurement. Far-UV spectra were recorded from 195 to 280 nm. Wavelength scans recorded ellipticity every 0.5 nm.

For thermal denaturation and renaturation, the ellipticity change at 222 nm was recorded. Temperature was increased from 5°C to 95°C, and then continuously decreased from 95°C to 5°C. Data were collected in 0.5°C steps. The temperature increased at 60°C/hr. The fraction of folded protein was calculated using the following equation^44^: 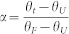


Here, α is the fraction folded, *θ_t_* is the observed ellipticity at any temperature, *θ_F_* is the ellipticity of the folded form, and *θ_U_* is the ellipticity of the unfolded form^44^. Data were plotted using the ORIGIN software.

## Supplementary Material

Supplementary Information Supplementary information

## Figures and Tables

**Table 1:** Sequence identity among designed *S*-HNLs, native *S*-HNL (*Me*HNL) and esterase (SABP2)

Enzyme name	SABP2^a^	HNL85	HNL54	HNL30	*Me*HNL
SABP2	100^b^	59.7	52.7	44.6	40.3
HNL85	59.7	100	84.8	68.6	63.2
HNL54	52.7	84.8	100	84.4	76.0
HNL30	44.6	68.6	84.4	100	82.2
*Me*HNL	40.3	63.2	76.0	82.2	100

^a^SABP2 is salicylic acid–binding protein 2 from *Nicotiana tabacum*.

^b^Sequence identity is represented in percent (%).

**Table 2:** Enzyme kinetic parameters of native (*Me*HNL), designed *S-*HNLs, and SABP2 toward racemic mandelonitrile^a^

Enzyme Name	*k*_cat_	*K*_m_	*k*_cat_/*K*_m_
	*min ^−1^*	*mM*	*min ^−1^ mM ^−1^*
SABP2 (G12T/M239K)^b^	0.9	13	0.072
HNL85	ND^c^	ND	ND
HNL54	12 ± 0.6^d^	3.2 ± 0.3	3.8
HNL30	156 ± 3	0.8 ± 0.1	195
*Me*HNL (WT)^a^	2070	5.2	398

^a^The parameters are cited from literature values reported by Asano *et al.*^20^.

^b^The parameters are cited from literature values reported by Padhi *et al.*^26^.

^c^ND means “not determined”.

^d^Parameters were calculated by performing experiments in triplicate. The parameters are represented as averages ± standard deviations.

**Table 3:** Steady-state parameters of three of the designed *S*-HNLs toward (*R*)- and (*S*)-mandelonitrile

	HNL85			HNL54			HNL30		
substrate	*k*_cat_ *min^−1^*	*K*_m_ *mM*	*k*_cat_/*K*_m_ *mM^−1^ min^−1^*	*k*_cat_ *min^−1^*	*K*_m_ *mM*	*k*_cat_/*K*_m_ *mM^−1^ min^−1^*	*k*_cat_ *min^−1^*	*K*_m_ *mM*	*k*_cat_/*K*_m_ *mM^−1^ min^−1^*
*(S)*-Man	ND	ND	ND	23.8	0.61	39.3	192	0.4	480
*(R)*-Man	ND^b^	ND	ND	3.0	0.37	8.1	18.0	5.0	3.6
*E* value^a^		ND			4.9 (*S*-selective)			133 (*S-*selective)	

^a^The *E* value was calculated by following the equation shown in reference^45^.

^b^ND means that the parameters could not be determined because of low enzymatic activity.
