# Supplementary material for: Protein evolution analysis of S-hydroxynitrile lyase by complete sequence design utilizing the INTMSAlign software
Source: Sci Rep. 2015 Feb 3;5:8193. doi: 10.1038/srep08193 (PMC4648443; doi:10.1038/srep08193)
Supplement: Supplementary Information [file srep08193-s1.pdf]

Supplementary Material

Protein evolution analysis of *S*-hydroxynitrile lyase by complete sequence design utilizing the INTMSAlign software

Shogo Nakano[1, 2] and Yasuhisa Asano[1, 2]

1, Biotechnology Research Center and Department of Biotechnology, Toyama Prefectural University, 5180 Kurokawa, Imizu, Toyama 939-0398, Japan

2, Asano Active Enzyme Molecule Project, ERATO, JST, 5180 Kurokawa, Imizu, Toyama 939-0398, Japan

The detailed algorithm of INTMSAlign

Schematic view of INTMSAlign was shown in Supplementary Figure 1. INTMSAlign uses two files: one contains sequence of target protein (STP) and another (library in Supplementary Fig. 1) contains sequences belonging to the same family as that of STP. The library is constituted by hundreds to thousands of primary sequences which are the family protein of STP. There are some approaches to prepare the library; i), selecting the sequences from Blastp web server [1] by submitting the STP, ii), from PubMed web server by inputting some keywords, such as EC number and protein name. After STP and the library were prepared, following two parameters have to be defined: N_{trial} and N_{pick}. INTMSAlign picks up one STP and N_{pick} number of sequences randomly from the library, and generates an ofile (Supplementary Fig.1). This process is repeatedly performed until the number of the ofiles reaches to total N_{trial} number (Supplementary Fig.1). For the all ofiles, MSA is performed by the program "CLUSTALW".

After all of MSA processes were completed, INTMSAlign generates total N_{trial} number of aln files (Supplementary Fig.1). INTMSAlign integrates all generated aln files by counting up a number of the appeared 20 amino acid residues and gaps based on each residue of STP. As for the details how to count up were shown in Supplementary Fig.2. Sum of the number of 20 amino acid residues and gap for *ith* residue of STP was defined as following:

$$N_{ij,raw} = (n_{i1}, n_{i2}, ..., n_{ij}, .., n_{i21})$$
$$\sum_{j=1}^{21} n_{ij} = (N_{pick} + 1) \times N_{trial} \quad \bullet\bullet\bullet(1)$$

Maximum value of "*i*" should be sequence length of STP. The *j* value corresponds to 20 amino acid residues (j = 1 ~ 20) and gap (j = 21). The *j* value is arranged in ascending order when one letter expression of amino acid residues is lined up in alphabetical order. For example, the *j* value is 1, 2, 3 and 20, then these represent Ala (A), Cys (C), Asp (D) and Tyr (Y), respectively.

Because the STP is included in every ofiles (Supplementary Fig.1), the number of amino acid residues of the STP is counted iteratively and reflected to the result file. This brings some bias, especially in case that N_{pick} number is small. Thus, to remove the bias, INTMSAlign subtracts N_{trial} number of the overcounted STP's sequence from $N_{ij,raw}$.

$$N_{ij} = N_{ij,raw} (j \neq x)$$
$$N_{ij} = N_{ij,raw} - k_{ix} (j = x)$$
$$k_{ix} = N_{trial} \quad \bullet\bullet\bullet(2)$$

After all of the process completed, appearance rate of amino acid residues are calculated for all residues of STP (Supplementary Fig.1) by following equation:

$$R_{ij} = \frac{N_{ij}}{(N_{trial}) \times (N_{pick} + 1) - k_{ix}}$$
$$\sum_{j=1}^{21} R_{ij} = 100\% \quad \bullet\bullet\bullet(3)$$

R_{ij} is calculated for 20 of all amino acid residues and gap; the R_{ij} represents the appearance rate at *ith* of the STP sequences of amino acid residues *(j)*. The R_{ij} is two dimensional arrays which have $i \times 21$ elements. The rate is saved as a text format data, which named result file (Supplementary

Fig.1).

Residue fixation: comparison of two result files to assign correlation residues

INTMSAlign has a function to calculate the appearance rate by only selecting primary sequences in the library which have the same amino acid residues (residue *X*) at certain residue number (l^{th} residue) as user defined one (*l:X*). In this study, we called the function “residue fixation” (Supplementary Fig.3). This could be used to perform the curation of the library.

In this section, to describe the residue fixation, we postulated the preparation of following two result files, Result-file A and Result-file B. In terms of the preparation, the identical STP and the library were utilized to prepare the two result files. Residues, which are highly conserved as different ones in each result file, could be regarded as correlation residues; appearance rate of the residues should be perturbed when MSA is performed by changing combination of primary sequences. These are corresponded with definition of correlation residues [2]. To extract correlation residues from the result files, D-score was defined as following:

$$D-score(i) = \frac{\sum_{j=1}^{20}\left[\left(R_{ij}\right)_A - \left(R_{ij}\right)_B\right]^2}{200} \quad \cdots(4)$$

Maximum D-score value would be 100, and the value is obtained in case that the *i*th residue is differently and perfectly conserved in two result files. Residues having high D-score value are correlation residues; the residues are correlatively mutated and highly conserved in different amino acid residues in the Result-file A and the Result-file B.

Assignment of consensus residues of MeHNL: Ser80, His236 and Lys237

The INTMSAlign could assign consensus residues accurately as well as other MSA program. To show this, appearance rate of two residues, Ser80 and His236, was calculated. Bar graph of appearance rate for the residues was shown in Supplementary Fig.3. Ser80 and His236 were highly conserved; appearance rates were 84.8% (Ser, Supplementary Fig.3A) and 89.2% (His, Supplementary Fig.3B) for 80th and 236th residues, respectively. The Ser80 and His236 are conserved in both *S*-HNL and the esterases, because these residues are catalytic residues for both enzymes [3].

Next, appearance rate for Lys237 was calculated. The 237th residue is known as the marker residue; the 237th residue is Lys in *S*-HNL and Met in the esterases, respectively [3]. From the analysis by INTMSAlign, the appearance rate of Lys was significantly low; the rate was 1.8% (Lys, Supplementary Fig.3C). On the other hand, the appearance rate about Met and Ser was significantly higher than Lys; the rate was 33.0 (Met) and 29.7% (Ser) (Supplementary Fig.3C), respectively. The high appearance rate of Met at the 237th residue should represent that the library seemed to be biased to the esterase family.

Assignment of correlation residues which brought difference of substrate specificity between S-HNLs and esterase

In this section, we will indicate that INTMSAlign can pick up the residues which are independently conserved in *S*-HNL and esterases, in a word, correlation residues. The 237th residue of *Me*HNL is the marker residue between *S*-HNL and esterases. With referring to this, two result files were generated using the residue fixation; one result file is generated by fixing the 237th residue as Lys (237:K) and another one is generated by fixing the 237th residue as Met (237:M). The former and the latter result files are generated with intend to represent amino acid appearance rate of only *S*-HNL and esterase, respectively. In fact, in the 237:K condition (*S*-HNL), residues 11th, 79th and 239th were highly conserved as Thr (84.9%), Glu (84.1%) and Gln (88.7%), respectively, and these residues were also conserved in *Me*HNL [4]. On the other hand, in the 237:M condition (esterase), residues 11th, 79th, and 239th were highly conserved as Gly (86.8%), His (90.4%) and Met (92.4%), respectively, and these were also conserved in SABP2 [5].

D-score was calculated for each residue of *Me*HNL to extract the correlation residue. Six residues which had D-score value more than 60.0 were picked up and shown in Supplementary Table 2. Among these residues, four residues (237th, 239th, 79th, and 11th) located on the active site are plotted in Supplementary Fig.5. The residue fixation of the INTMSAlign works correctly because the appearance rate at 237th residue was 100% in cases of using two result files of which conditions are the 237:K (black bar, Supplementary Fig.5A) and 237:M (red bar, Supplementary Fig.5A), respectively.

Here, 11th residue of *Me*HNL had the 5th highest D-score value (64.0, Supplementary Table 2) in

all of the residues. The 11th residue was Thr in *S*-HNL (black bar in Supplementary Fig.5D) and Gly in esterase (red bar, Supplementary Fig.5D), respectively. In SABP2, the 11th residue corresponds to Gly12, and this is one of two residues which are important to have *S*-HNL activity [3]. Residue 79 had the 4th highly D-score value (66.7, Supplementary Table 2); the residue is conserved in Glu in *S*-HNL and His in esterase, respectively (Supplementary Fig.5C). The Glu79 is one of active site residues of *S*-HNL; enzyme reactivity of the *Me*HNL (E79A) loses to 1% of that of *Me*HNL (WT) [6].

To show where these residues are located, four residues (Thr11, Glu79, Lys237 and Gln239) of *Me*HNL (Supplementary Table 2) are shown in Supplementary Fig.6A. The residues are located near active site of *Me*HNL, and formed hydrogen bond network each other (Supplementary Fig.6A). As for residue 198 and 219 in Supplementary Table 2, these are located at remote position from the active site (Supplementary Fig.6B). Remotely located residues from active site often regulates dynamics of proteins to achieve efficient enzyme catalysis [7], and, therefore, these two residues may control the dynamics of *S*-HNL and esterase to express their function.

Figure Legends

Supplementary Figure 1, The algorithm of INTMSAlign. In STP, there is one sequence of target protein, and in library, there are some sequences of family protein of the target protein. There is no limit to the number of sequences in library.

Supplementary Figure 2, Description of how to count up a number of amino acid residues by INTMSAlign. Using the STP sequence as a basis, a number of amino acid residues are counted up. In this figure, 1st, 2nd and 3rd residues should be Met, Ala and Ser, respectively, and gap inserted in STP by the MSA (represents as "-") is skipped. After counting up the number of the amino acid residues completed, amino acid appearance rate was calculated. Before the calculation, a number of residues derived from STP sequence are subtracted. For example, 2nd column of the figure, number of Ala, Ser, Thr and sum of the residues are 2-1 = 1, 1, 3 and 6-1 = 5, respectively. The subtraction was performed to remove a bias which brought by over counting a number of STP sequence iteratively with progress of the INTMSAlign process.

Supplementary Figure 3, The appearance rate at Ser80 (A), His236 (B) and Lys237 (C) of *Me*HNL. The x and y axis represents amino acid residues and appearance rate, respectively. The figures were drawn by ORIGIN.

Supplementary Figure 4, Description of residue fixation of the INTMSAlign. In this figure, we hypothesized that this is one of the alnfile (Fig. 1), and regarded LIB1, 2, 4, 9 and 13 as picked sequences from the library. By defining "*2:T*", INTMSAlign will select three sequences (LIB4, 9, 13), which have Thr at the 2rd position of the STP sequence and colored by red in the figure, and calculate amino acid appearance rate.

Supplementary Figure 5, The appearance rate at residue 237 (A), 239 (B), 79 (C) and 11 (D) of *Me*HNL. The data which were obtained by fixing residue 237 as Lys (237:K) and Met (237:M) were represented by black and red bar, respectively.

Supplementary Figure 6, The structure of *Me*HNL (PDB ID: 1EB9). A), the location of residue 79 and 239. The two residues were colored by orange, and four catalytic residues (Thr11, Ser80, His236 and Lys237) were colored by green. **B) the location of residue 198 and 219.** They located at far from active site. The Figure was drawn by program PyMol [8].

Supplementary Figure 7, Multiple sequence alignment among artificial *S*-HNLs, native *S*-HNL (*Me*HNL), and esterase (SABP2). The active site residues (Thr11, Ser80, Asp208 and Lys237) were shown as asterisk (*). The residues which relate to improve solubility in *E.coli*

expression system (His103, Lys176, Lys199 and Lys224) were shown as arrowhead. Completely conserved residues among the five sequences were highlighted by filled-red color.

Supplementary Figure 8, Non-rooted (A) and rooted (B) phylogenetic trees of the three artificial *S*-HNLs and 11 sequences of α/β hydrolase fold family. The phylogenetic tree was built by program MEGA6 [9] utilizing maximum likelihood method. The number written near node was bootstrap value, and the value was obtained from 1000 resampling. The name and accession code of each protein in the trees are as followings: TcSABP2 is salicylic acid-binding protein 2 from *Theobroma cacao* (XP_007047144.1), PpHyp is hypothetical protein from *Prunus persica* (XP_007202442.1), SABP2 is salicylic acid-binding protein 2 from *Nicotiana tabacum* (Q6RYA0.1), TcMetest is methyl esterase 10, putative from *Theobroma cacao* (XP_007047146.1), GmHyp is uncharacterized protein LOC100803613 from *Glycine max* (NP_001239778.1), AtMetest4 is methyl esterase 4 from *Arabidopsis thaliana* (NP_179939.1), AtHNL is methyl esterase 5 from *Arabidopsis thaliana* (NP_196592.1), Eshyp is hypothetical protein EUTSA_v10014451mg from *Eutrema salsugineum* (XP_006399524.1), BmHNL is *(S)*-hydroxynitrile lyase from *Baliospermum montanum* (BAI50630.1), MeHNL is *(S)*-hydroxynitrile lyase from *Manihot esculenta* (P52705.3), HbHNL is *(S)*-hydroxynitrile lyase from *Hevea brasiliensis* (P52704.1).

Supplementary Figure 9, Enzyme concentration dependent HPLC profile of *(R)*- and *(S)*-Man production by artificial *S*-HNLs (HNL85, HNL54 and HNL30). The enzyme concentration of the added HNL85 (A), HNL54 (B) and HNL30 (C) were 47.9, 7.17, and 0.47 mg/mL, respectively. The red, blue and magenta lines were obtained by adding 10, 50, and 100 μL of the artificial *S*-HNLs solution to the reaction buffer. Peak area belonging to (R)-Man was larger with increase of the concentration in HNL85 and HNL54 (A and B). On the contrary to this, the area was smaller in HNL30 (C)

Table contents

Supplementary Table 1. Parameters of the INTMSAlign

	*Me*HNL
Types of Blast	Blastp
Database	Non redundant
Sequence of target protein (STP)	*Me*HNL (genbank ID:55469815)
Total number of sequences in the library	823[*3]
N_{pick}	8
N_{trial}	1000
Residue fixation (*S*-HNL)[*1]	237:K[*2]
Residue fixation (esterase)[*1]	237:M[*2]

*1, The 237th residue was conserved as Lys and met in *S*-HNL and esterase, respectively.

*2, 237:K and 237:M represent that the INTMSAlign only selects sequences which have Lys and Met at 237th residue, respectively.

*3, Accession code of all sequences in the library was shown in Supplementary Table 3.

Supplementary Table 2 D-score values which calculated by comparing HNL with esterase.

Residue Number[*1]	D-score	Residue in HNL	Residue in esterase
237	100	Lys	Met
239	77.3	Gln	Met
198	72.0	Ile	Val
79	66.7	Glu	His
11	64.0	Thr	Gly
219	61.4	Gln	Met

*1, To calculate the D-scores, we fixed 237th residue as Lys and Met, respectively, and obtained these data.

Supplementary Table 3. Accession code of total 823 sequences in the library.

460401605, 449448524, 293337149, 115442069, 302799778, 302768901, 218189682, 125534438, 359496072, 460395450, 297720733, 474407269, 326488669, 326512058, 414880181, 413918007, 475578874, 413918006, 226506656, 222618414, 115442067, 242059801, 460372674, 482564101, 297815322, 116783632, 502167619, 502167607, 242059809, 482564799, 449455222, 357512897, 302792114, 116793113, 473758751, 326516868, 460395456, 470136902, 242080259, 475526435, 508706269, 474150162, 334184394, 125577187, 224035639, 116784948, 502170453, 334184392, 116780076, 226491548, 482564637, 413921497, 388520287, 302784178, 302803552, 356568777, 388516841, 357144410, 15230077, 449438717, 4006863, 225460275, 3242718, 218185126, 115483865, 255585722, 514735749, 224141085, 222616517, 462411685, 297612550, 334187239, 297825261, 470129289, 10086476, 357157580, 224077736, 475598292, 302813798, 297796743, 356516217, 218186310, 125601948, 115474437, 125559901, 460386716, 242067261, 475610595, 514791969, 388510928, 514807330, 460369959, 115473231, 359488571, 514805054, 508709774, 388504308, 15237165, 473887440, 462415255, 15218463, 460390443, 21595837, 414887498, 242046208, 470122393, 71534876, 147771160, 357122066, 168012855, 302795029, 356509098, 482559984, 357512921, 125600998, 514805052, 125559095, 326516130, 508709775, 255562689, 356540703, 224128027, 502127085, 225448733, 255539408, 226533134, 449447422, 470117134, 470144193, 508699406, 116779412, 297833870, 460392565, 508728297, 168040830, 15228391, 297845568, 482558392, 224035423, 226493396, 356517822, 474412065, 12325081, 4538993, 42563068, 125552074, 6730643, 226497974, 482575198, 42562323, 482569120, 297841655, 473781236, 168058255, 449447611, 297813423, 224146598, 508779612, 168024125, 356509495, 118487370, 326492958, 508711814, 168022859, 508779613, 115485575, 462401325, 462419960, 302807967, 224145116, 449447511, 224135701, 482566909, 470116701, 118487976, 449520439, 222619825, 147842033, 30681236, 222631350, 482548390, 255635198, 225423454, 302785065, 462419345, 255542004, 168005002, 502136736, 297738098, 475611474, 514751191, 212274909, 302785073, 224171844, 218186309, 15866583, 54287491, 218185125, 168019981, 359496076, 115487020, 494995921, 147794582, 255636352, 222615402, 168044120, 475620379, 414588743, 224098992, 482553045, 474076929, 482573951, 297846378, 515819213, 460379398, 255633266, 15235844, 357153058, 356509100, 168055767, 226508846, 227206238, 168033458, 297805950, 297800448, 506991453, 297727915, 351723107, 475568793, 446031308, 122209128, 384185843, 507051310, 168064746, 446031309, 388514029, 56752301, 488117817, 302795472, 414878821, 488055444, 488097241, 488014848, 487938634, 222631348, 297736465, 489280920, 495609573, 446031306, 446031456, 357455487, 302796757, 446031455, 488064249, 163939739, 446031460, 218233112, 515927872, 158523175, 488086922, 491178901, 413951380, 488114743, 446031458, 488077413, 446031457, 339328685, 489065481, 407704309, 507020715, 515993990, 238025578, 507032828, 446031462, 505587800, 446031459, 487992696, 494761035, 255597864, 507063357, 473948166, 226188032, 507022183, 489066646, 77552851, 356522980, 77548461, 495622169, 148807137, 491290746, 446031463, 255559729, 507013916, 475610594, 297735845, 495069631, 297735846, 497515626, 492483188, 475598293, 449528258, 496458611, 517114025, 330822056, 495089240, 474412064, 242067259, 496583585, 169647185, 7672985, 497868664, 9797761, 517444413, 521460719, 113473786, 375145080, 492824147, 518882952, 356502231, 255562685, 334345734, 489402080, 489424168, 515871861, 497873715, 475540803, 56709214, 518788533, 495134131, 110681069, 497824997, 125573231, 473865068, 224148559, 356496844, 496158607, 514791965, 339502071, 497492947, 518800609, 300311758, 224096836, 518126990, 495830450, 497980682, 255556247, 494383542, 358348240, 402570311, 498123820, 495504524, 515900333, 489394316, 497796695, 497380824, 520866780, 162448964, 517883650, 255562683, 56544478, 497191963, 289164340, 491146801, 489373384, 297741340, 242053253, 148553931, 515815086, 497803388, 298291541, 146283233, 518235695, 386021656, 517201941, 497510940, 489387654, 496032449, 392422081, 495838276, 53715878, 126445915, 83716578, 490680700, 497587661, 515117267, 490306154, 121597836, 53723358, 126444466, 490683279, 496368305, 497636878, 492453876, 76817876, 403524387, 518640270, 482564934, 491140581, 126457990, 490664559, 497627126, 482568548, 339495015, 83645824, 397687944, 494082072, 255556249, 518084952, 491128892, 495622272, 497601964, 492908015, 508709776, 120404337, 474389263, 493803817, 374983815, 403529254, 495178976, 115359700, 256397969, 347527657, 493819171, 172064517, 516022866, 167646355, 331698066, 493566349, 521092280, 518730658, 497774739, 522125869, 389864520, 226311396, 475548483, 516817135, 351721677, 519066162, 357387418, 257057778, 497257814, 475508653, 488612323, 516613077, 515816758, 170751104, 490029446, 34394562,

516325389, 517517015, 387904213, 119962164, 374985555, 431926552, 470176733, 78060868, 126435607, 518486937, 493460904, 392417421, 161519720, 377811670, 494778668, 224112106, 495000693, 493449348, 518898864, 145223063, 493442115, 507103268, 521988580, 489177059, 134099834, 497496849, 284991870, 493258567, 517339650, 134292516, 493439760, 495108244, 50083981, 315443520, 515110091, 120405131, 518322202, 517609183, 489172941, 271968469, 427719439, 519331847, 494688576, 271966963, 518985997, 374312610, 512488567, 497402756, 331697487, 493526162, 255556251, 490072416, 121603139, 21222782, 516553615, 515764713, 206564672, 389872196, 91199646, 490025761, 315444485, 488084365, 182440665, 383779210, 145224150, 495637362, 522162173, 146278282, 379735998, 517361273, 517899945, 326786736, 515116022, 257129265, 345003428, 116693776, 518865497, 107027512, 170736922, 517388915, 108799979, 494482933, 496444820, 490588134, 490591998, 490556954,

References

1. Altschul SF, *et al.* Gapped BLAST and PSI-BLAST: a new generation of protein database search programs. *Nucleic acids research* **25**, 3389-3402 (1997).

2. Magliery TJ, Lavinder JJ, Sullivan BJ. Protein stability by number: high-throughput and statistical approaches to one of protein science's most difficult problems. *Current opinion in chemical biology* **15**, 443-451 (2011).

3. Padhi SK, Fujii R, Legatt GA, Fossum SL, Berchtold R, Kazlauskas RJ. Switching from an esterase to a hydroxynitrile lyase mechanism requires only two amino acid substitutions. *Chemistry & biology* **17**, 863-871 (2010).

4. Asano Y, Dadashipour M, Yamazaki M, Doi N, Komeda H. Functional expression of a plant hydroxynitrile lyase in Escherichia coli by directed evolution: creation and characterization of highly in vivo soluble mutants. *Protein Engineering Design & Selection* **24**, 607-616 (2011).

5. Forouhar F*, et al.* Structural and biochemical studies identify tobacco SABP2 as a methyl salicylate esterase and implicate it in plant innate immunity. *Proceedings of the National Academy of Sciences of the United States of America* **102**, 1773-1778 (2005).

6. Hasslacher M, Kratky C, Griengl H, Schwab H, Kohlwein SD. Hydroxynitrile lyase from Hevea brasiliensis: molecular characterization and mechanism of enzyme catalysis. *Proteins* **27**, 438-449 (1997).

7. Lee J, Goodey NM. Catalytic contributions from remote regions of enzyme structure. *Chemical reviews* **111**, 7595-7624 (2011).

8. Delano W, L. The PyMOL molecular graphics system. (ed^(eds Delano Scientific SC, CA.) (2002).

9. Tamura K, Stecher G, Peterson D, Filipski A, Kumar S. MEGA6: Molecular Evolutionary Genetics Analysis version 6.0. *Molecular biology and evolution* **30**, 2725-2729 (2013).

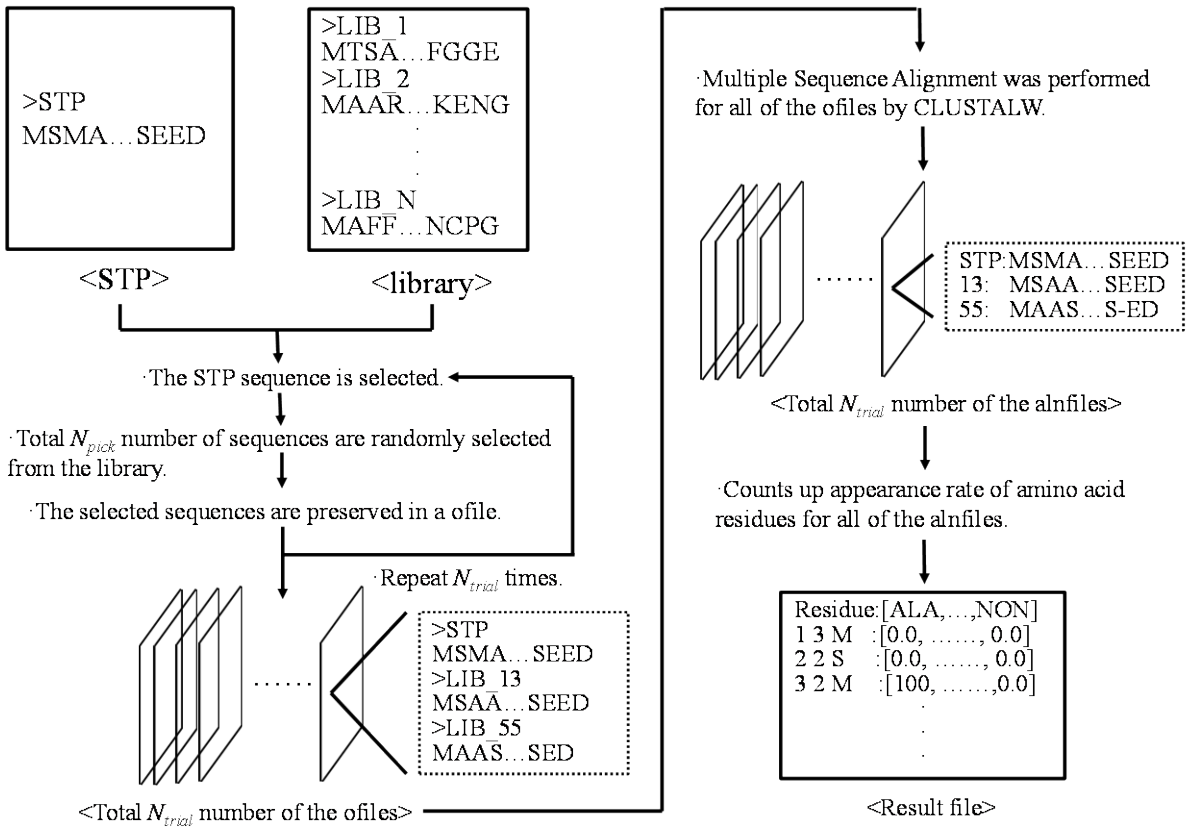

Supplementary Figure 1

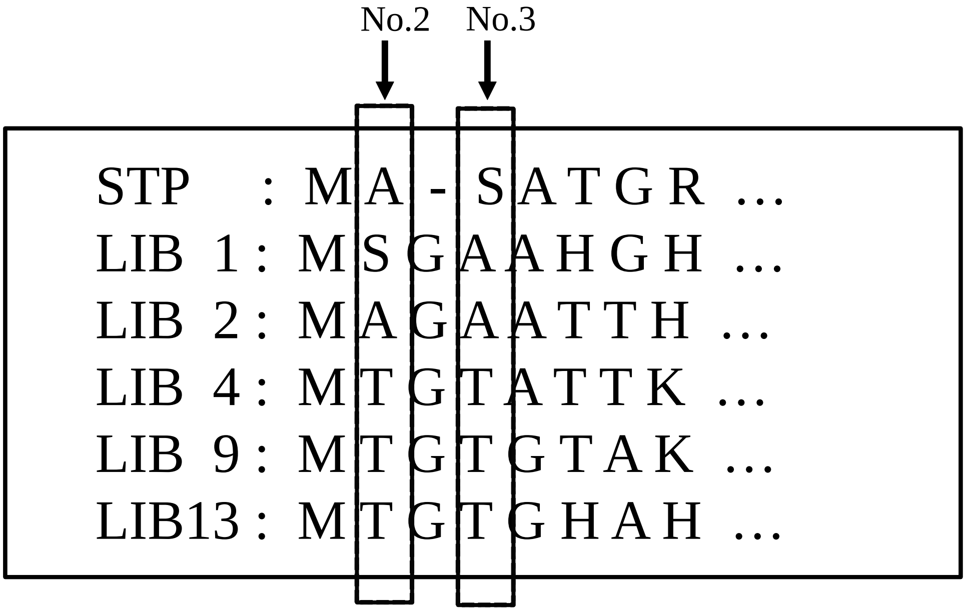

[Result]
1 M : 0.0 (Ala), …, 100.0 (Met), …, 0.0 (Non) → (No.1 Met:6-1 = 5)
2 A : 20.0 (Ala), …, 20.0 (Ser), 60.0 (Thr), …, 0.0 (Non) → (No.2 Ala:2-1 = 1, Ser:1, Thr:3)
3 S : 40.0 (Ala), …, 60.0 (Thr), … 0.0 (Non) → (No.3 Ala:2, Ser:1-1 = 0, Thr:3)

Supplementary Figure 2

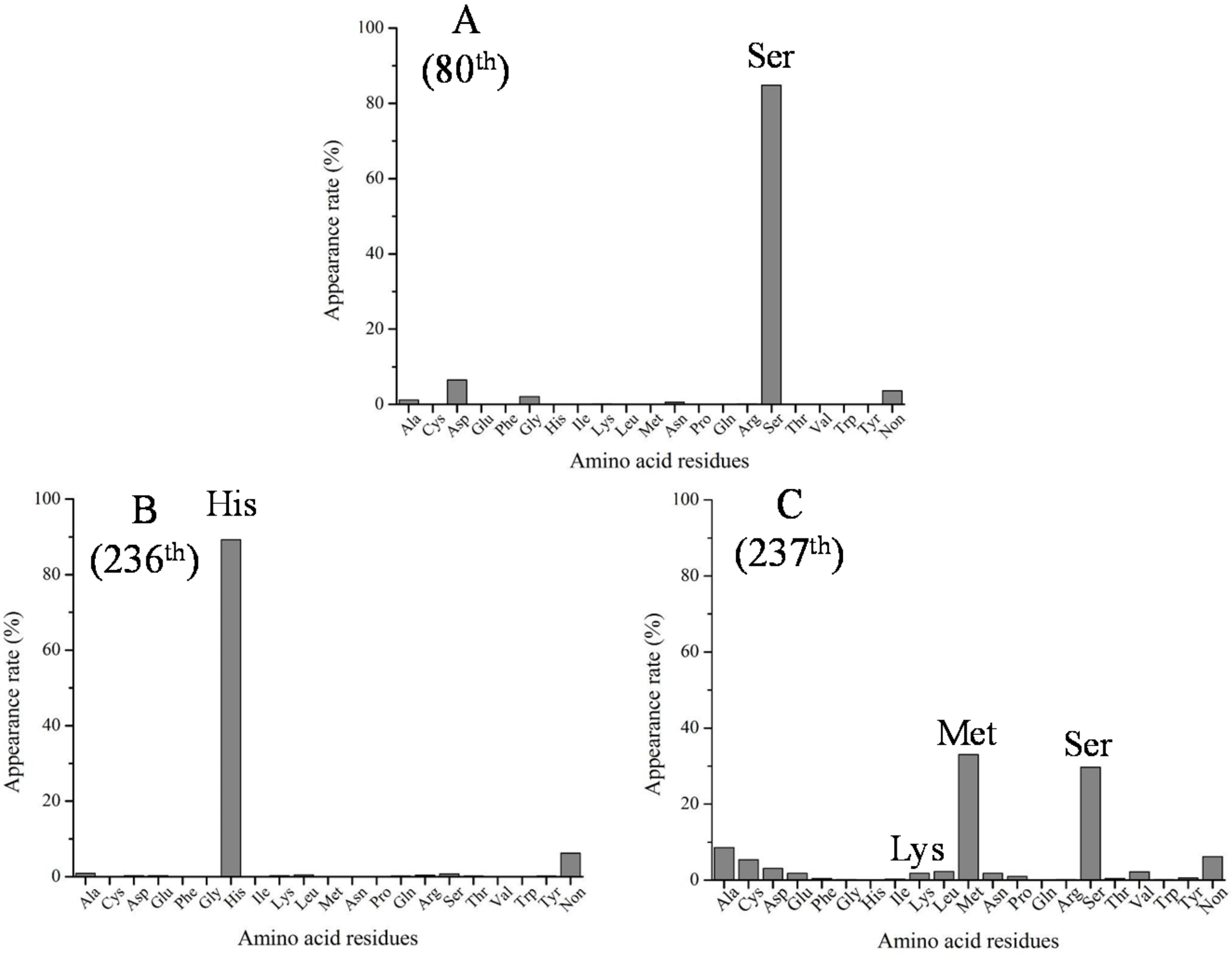

Supplementary Figure 3

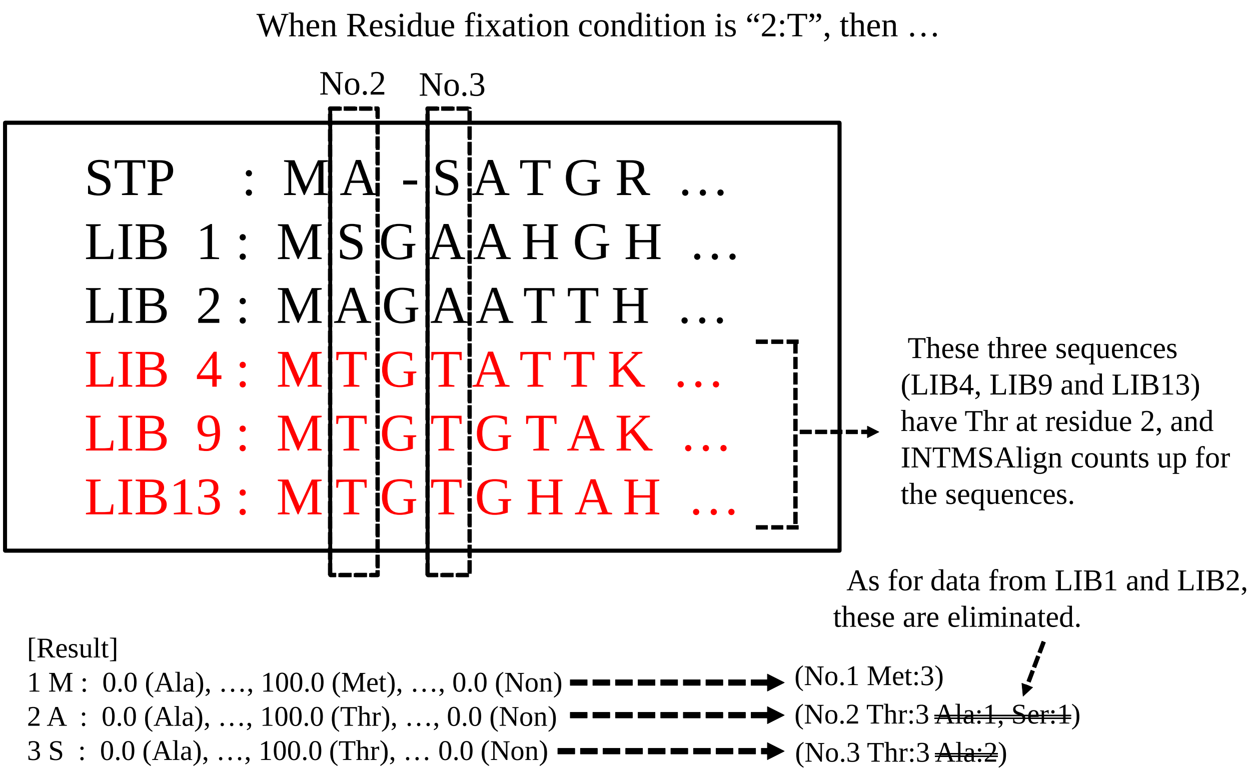

Supplementary Figure 4

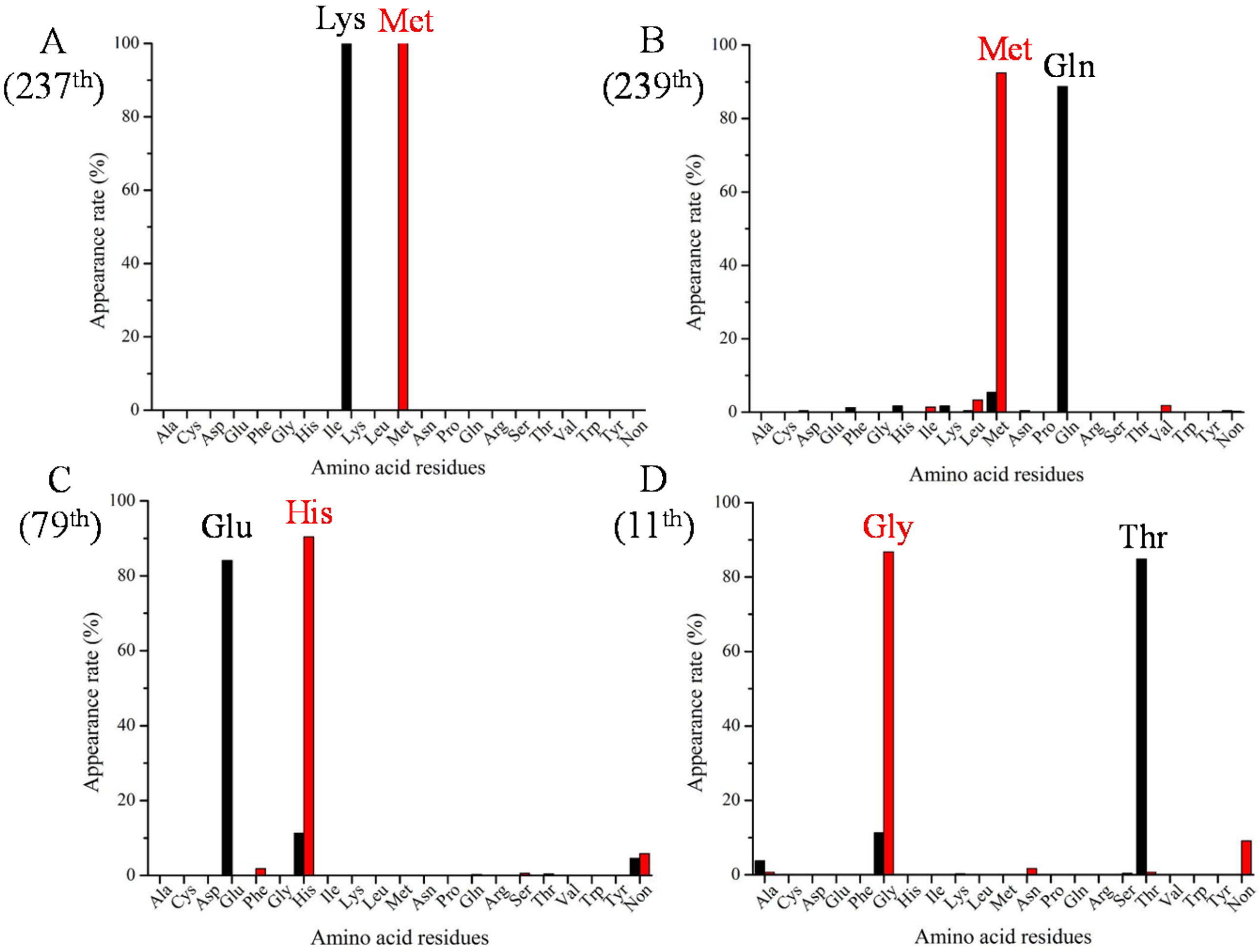

Supplementary Figure 5

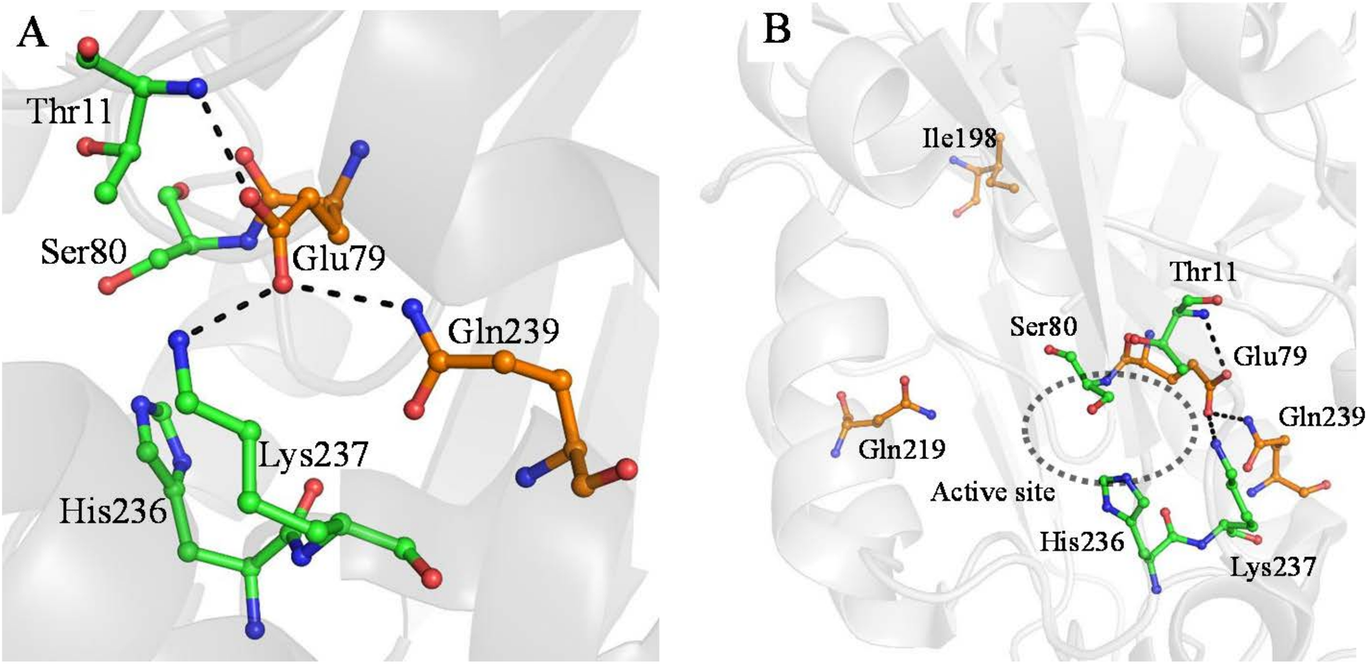

Supplementary Figure 6

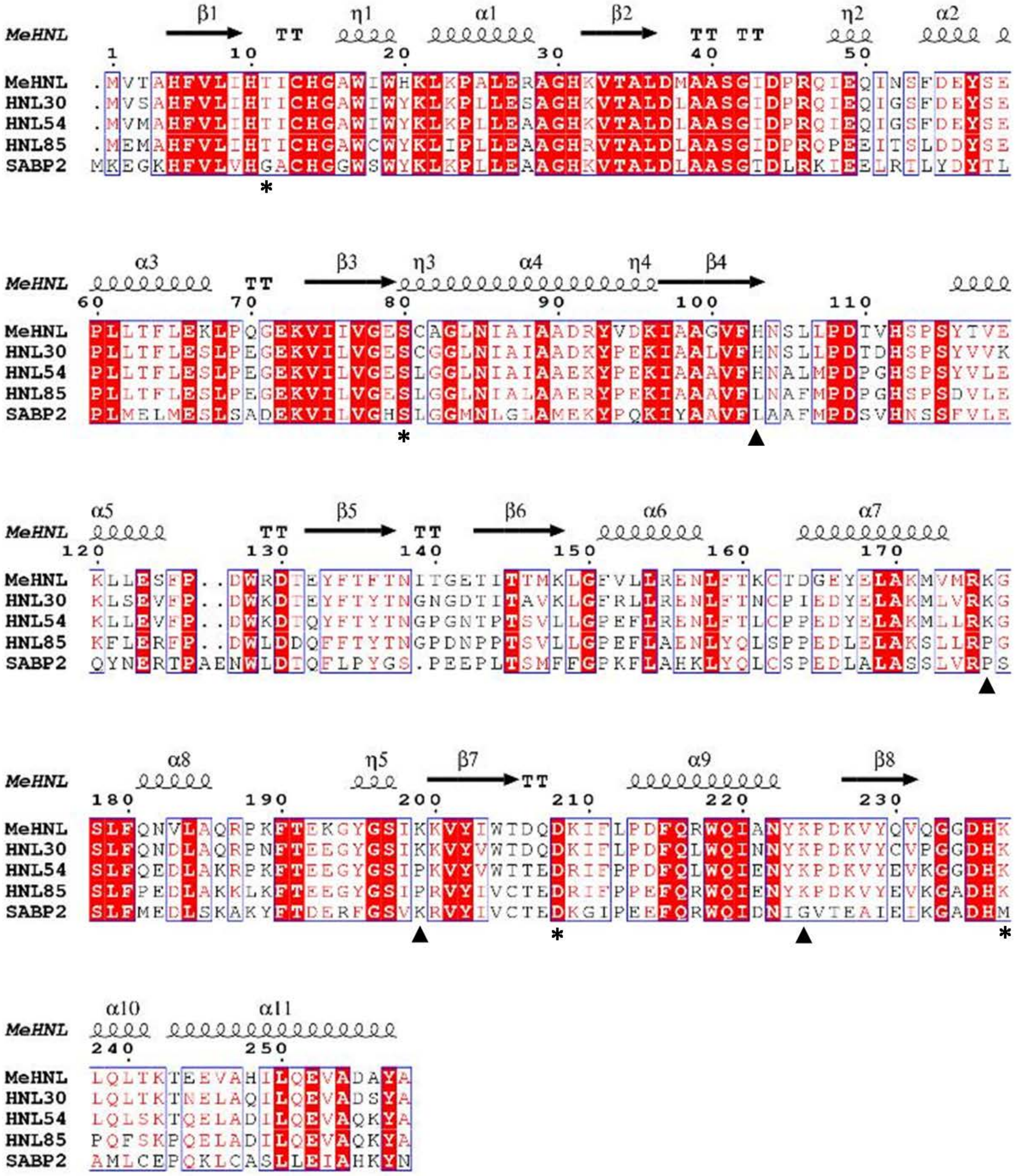

Supplementary Figure 7

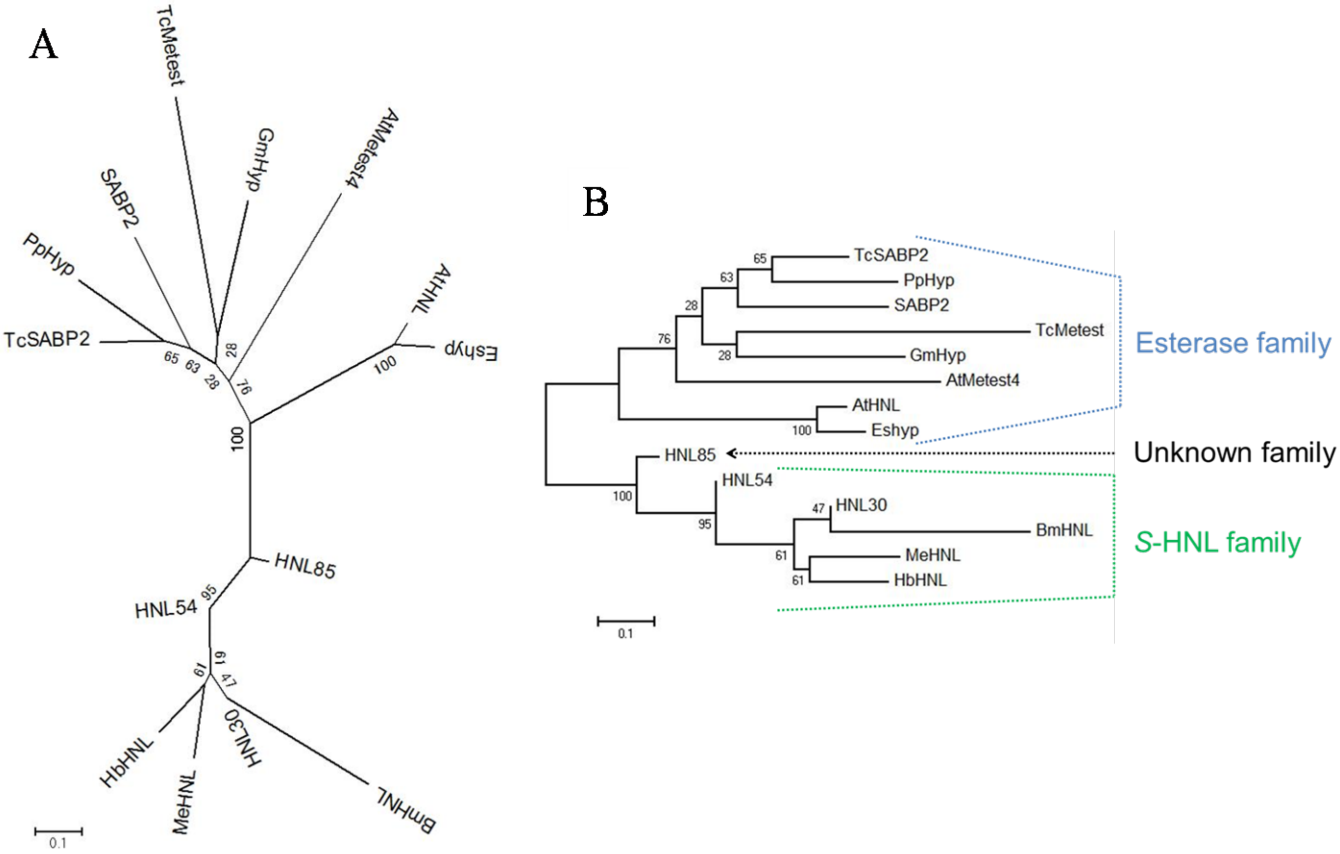

Supplementary Figure 8

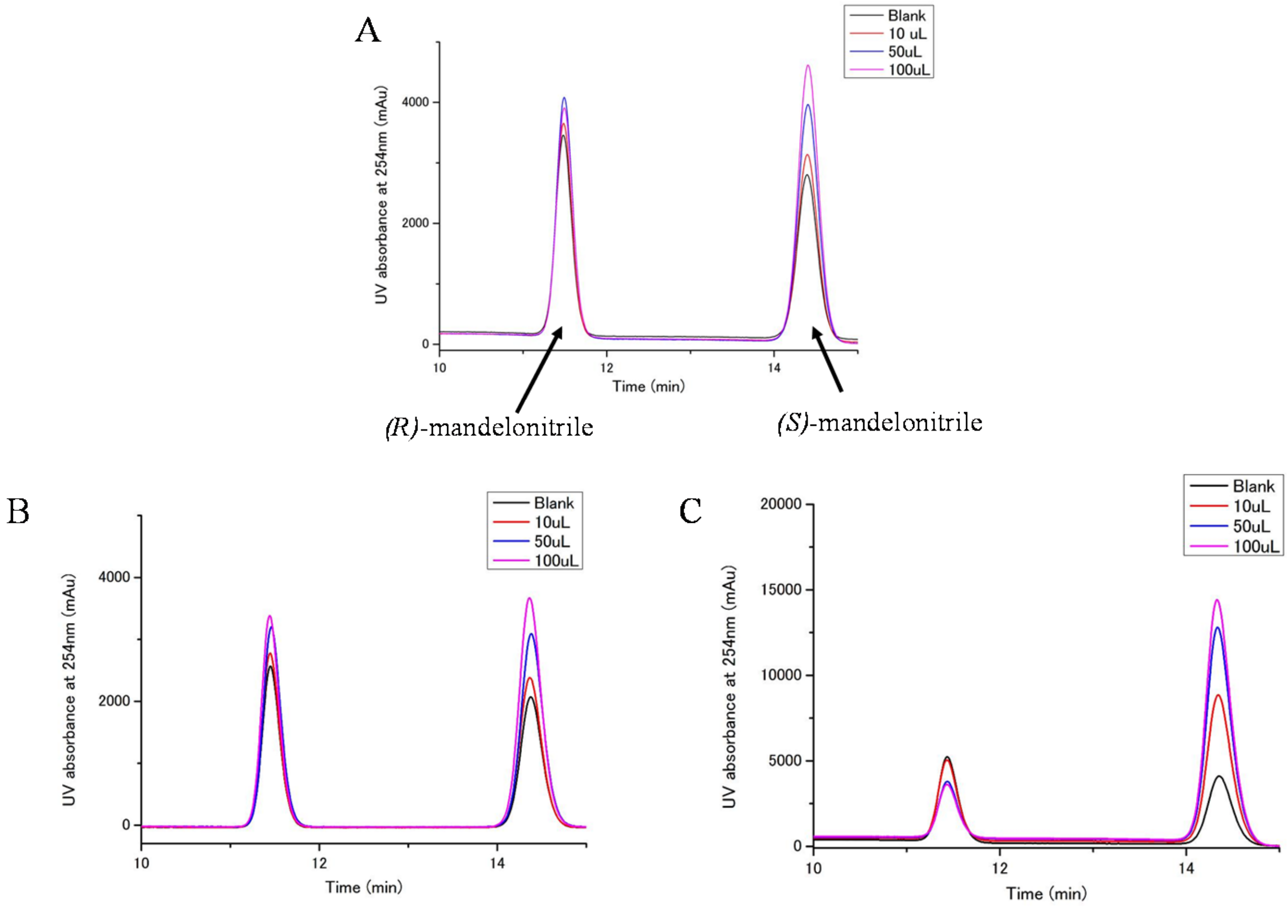

Supplementary Figure 9
